# Akebia saponin D protects hippocampal neurogenesis from microglia-mediated inflammation and ameliorates depressive-like behaviors and cognitive impairment in mice through the PI3K-Akt pathway

**DOI:** 10.3389/fphar.2022.927419

**Published:** 2022-08-30

**Authors:** Qin Liu, Jinqiang Zhang, Chenghong Xiao, Dapeng Su, Liangyuan Li, Changgui Yang, Zhihuang Zhao, Weike Jiang, Zili You, Tao Zhou

**Affiliations:** ^1^ Resource Institute for Chinese & Ethnic Materia Medica, Guizhou University of Traditional Chinese Medicine, Guiyang, China; ^2^ School of Life Science and Technology, University of Electronic Science and Technology of China, Chengdu, China

**Keywords:** neuroinflammation, akebia saponin D, cognitive impairment, depression, neurogenesis, microglia, neural stem/precursor cell, PI3K-Akt signaling pathway

## Abstract

Given the ability of akebia saponin D (ASD) to protect various types of stem cells, in the present study, we hypothesized that ASD could promote the proliferation, differentiation, and survival of neural stem/precursor cells (NSPCs), even in a microglia-mediated inflammatory environment, thereby mitigating inflammation-related neuropsychopathology. We established a mouse model of chronic neuroinflammation by exposing animals to low-dose lipopolysaccharide (LPS, 0.25 mg/kg/d) for 14 days. The results showed that chronic exposure to LPS strikingly reduced hippocampal levels of PI3K and pAkt and neurogenesis in mice. In the presen of a microglia-mediated inflammatory niche, the PI3K-Akt signaling in cultured NSPCs was inhibited, promoting their apoptosis and differentiation into astrocytes, while decreasing neurogenesis. Conversely, ASD strongly increased the levels of PI3K and pAkt and stimulated NSPC proliferation, survival and neuronal differentiation in the microglia-mediated inflammatory niche *in vitro* and *in vivo*. ASD also restored the synaptic function of hippocampal neurons and ameliorated depressive- and anxiety-like behaviors and cognitive impairment in mice chronically exposed to LPS. The results from network pharmacology analysis showed that the PI3K-AKT pathway is one of the targets of ASD to against major depressive disorder (MDD), anxiety and Alzheimer’s disease (AD). And the results from molecular docking based on computer modeling showed that ASD is bound to the interaction interface of the PI3K and AKT. The PI3K-Akt inhibitor LY294002 blocked the therapeutic effects of ASD *in vitro* and *in vivo*. These results suggested that ASD protects NSPCs from the microglia-mediated inflammatory niche, promoting their proliferation, survival and neuronal differentiation, as well as ameliorating depressive- and anxiety-like behaviors and cognitive impairment by activating the PI3K-AKT pathway. Our work suggests the potential of ASD for treating Alzheimer’s disease, depression and other cognitive disorders involving impaired neurogenesis by microglia-mediated inflammation.

## Highlights

1) ASD protects NSPCs from the microglia-mediated inflammatory niche and stimulates NSPC proliferation and neuronal differentiation.
2) The PI3K-Akt pathway helps mediate the neuroprotective effects of ASD.
3) ASD shows preclinical potential for treatment of disorders involving impaired neurogenesis

## 1 Introduction

Hippocampal neurogenesis plays an important role in structural plasticity and network maintenance in adults (Cope & Gould, 2019; Gage, 2021). Perturbation of adult neurogenesis contributes to several human diseases, including depression, anxiety, cognitive impairment and neurodegenerative diseases (Snyder et al., 2011; Anacker & Hen, 2017; Zhang J. et al., 2021; Terreros-Roncal et al., 2021). During aging, hippocampal neurogenesis declines, reducing stress resistance and increasing the risk of mood disorders and progression of cognitive impairment (Deng et al., 2010; Snyder et al., 2011; Mehdipour et al., 2021). For example, as mice age, the rate at which new granular cells form in the subgranular zone (SGZ) decreases around 40-fold (Morgenstern et al., 2008).

Neurogenesis in the adult brain can mitigate the effects of aging and neurodegeneration involving amyloid-β (Cameron & Glover, 2015; Bonafina et al., 2019). This implies that promoting neurogenesis in the adult hippocampus may be an effective treatment against Alzheimer’s disease and major depressive disorder (Berger et al., 2020).

Adult hippocampal neurogenesis is supported by the proliferation and differentiation of neural stem/precursor cells (NSPCs), which can differentiate into neurons, oligodendrocytes, and astrocytes, thereby counteracting the loss of neurons during aging (Bhattarai et al., 2016; Hattiangady et al., 2020). Such neurogenesis occurs mainly in the SGZ of the dentate gyrus (DG) and in the subventricular zone (SVZ) adjacent to the lateral ventricles (Drew et al., 2013; Jurkowski et al., 2020). Therefore, targeting the proliferation, differentiation, and survival of NSPCs in these zones may alleviate Alzheimer’s disease and major depressive disorder.

A challenge to maintaining NSPC proliferation and differentiation is the microglia-mediated neuroinflammatory niche (Sato, 2015). As innate immune cells, microglia can be activated by amyloid-β, cell debris, myelin, and other harmful substances, which enhance their phagocytic activity and thereby maintain brain homeostasis (Pap et al., 2021). However, microglia appear to be hyperactivated in patients and animal models of Alzheimer’s disease and major depressive disorder (Qiao et al., 2021). Microglia-mediated inflammation inhibits neurogenesis in the hippocampus of such patients (Santos et al., 2016; Hanslik & Ulland, 2020; Perea et al., 2020) by inhibiting NSPC proliferation and differentiation (Araki et al., 2021).

Researchers have found that akebia saponin D (ASD), a triterpenoid saponin extracted from Dipsacus asper Wall, a traditional Chinese medicine, induces the proliferation and differentiation of various types of stem cells as well as angiogenesis under inflammatory conditions (Ding et al., 2019; Gu et al., 2020; Niu et al., 2021; Chen et al., 2022). Several studies have shown that ASD can cross the blood-brain barrier to improve AD pathology and Aβ-induced cognitive deficits, as well as inflammation-induced depression (Yu et al., 2012; Wang et al., 2018; Zhang et al., 2020c; Stepnik, 2021). However, the molecular mechanisms by which ASD exerts these effects remain elusive. Based on the protective and promoting effects of ASD on the function of various stem cells, we wondered whether ASD might promote NSPC proliferation and differentiation, even in a microglia-mediated inflammatory environment. This led us to wonder whether ASD might exert its neurotherapeutic effects by robustly stimulating NPSCs in the hippocampus.

Therefore we evaluated the effects of ASD on hippocampal neurogenesis and behavior in a mouse model of chronic neuroinflammation. We also evaluated the effects of ASD on NSPC proliferation, differentiation, and survival in a microglia-mediated inflammatory niche *in vitro*. Our studies provide a mechanistic rationale for deepening the exploration of ASD as a promising treatment against Alzheimer’s disease, depression and other cognitive disorders involving impaired neurogenesis.

## 2 Material and methods

### 2.1 Network pharmacology analysis

To screen the pathways which ASD targets for major depressive disorder (MDD), anxiety and Alzheimer’s disease (AD), the network pharmacology analysis was performed based on previous studies (Zhang et al., 2021). In brief, potential MDD-, anxiety- and AD-related targets were retrieved from the Human Gene Database (GeneCards, https://www.genecards. org/) and the DisGeNET Database (https://www.disgenet.org/home/). Targets of ASD were obtained from the TCMSP and SwissTargetPrediction databases (http://www.Swisstargetprediction.ch). Then, the targets were standardized in the UniProt (https://www.uniprot.org) database with status set as ‘reviewed’ and species set as ‘human’. After removing duplicates, a database of ASD and their targets was constructed. Screened targets of the ASD and potential MDD-, anxiety- and AD-related targets were imported into a Venn diagram webtool (http://bioinformatics.psb.ugent.be/webtools/Venn/) for analysis, and common targets were extracted for further Kyoto Encyclopedia of Genes and Genomes (KEGG) pathway enrichment analysis using R software, a free software environment for statistical computing and graphics. The KEGG pathway analyses were screened for *p* < 0.05. The top 20 items of KEGG analysis identified from R software were mapped as bubble plots.

### 2.2 Molecular docking

The Surflex-Dock module of Sybyl-X2.1 software (Tripos Associates Inc., S.H.R.; St. Louis, MO 631444, USA) was used for molecular docking to predict the binding mode of ASD with the Pi3k and AKT based on previous studies (Yu et al., 2022).

### 2.3 Animals

Male C57BL/6J mice (7–8 weeks old) were purchased from Changsha Tianqin Biotechnology (Changsha, China) and caged individually. The mice were habituated to their new environment for 1 week. The mice were then habituated to a 1% sucrose solution for 48 h. Sucrose preference was tested before the experiment began, defined as day 0, and this preference as well as the animal body weight served as the baseline. The mice were then divided into seven groups as described in sections 2.4. All experiments were approved by the Institutional Animal Care and Use Committee of the Guizhou University of Traditional Chinese Medicine.

### 2.4 Pharmacological treatments *in vivo*

ASD (99.92% pure) was purchased from Chengdu Alfa Biotechnology (Chengdu, China). Lipopolysaccharide (LPS; Sigma-Aldrich, MO, USA) or ASD was dissolved in 0.9% saline at a concentration of 4 mg/ml. Minocycline hydrochloride (MedChemExpress, WeVoice, USA) or LY294002 (MedChemExpress) was dissolved to a concentration of 2.5 g/ml in 0.9% saline containing 5% dimethyl sulfoxide (DMSO). Mice were habituated to 1% sucrose solution for 1 week, then allocated into seven groups such that baseline sucrose preference and body weight did not differ significantly across the groups. The seven groups were as follows: control + vehicle (Ctrl), 0.25 mg/kg/d LPS + vehicle (LPS), LPS +10 mg/kg/d ASD [LPS + ASD (10 mg)], LPS +50 mg/kg/d ASD [LPS + ASD (50 mg)], LPS +100 mg/kg/d ASD [LPS + ASD (100 mg)], LPS +50 mg/kg/d minocycline hydrochloride (LPS + Mino), and LPS +100 mg/kg/d ASD +6 μg LY294002 (LPS + ASD + LY294002).

Mice were intraperitoneally administered vehicle, ASD, minocycline hydrochloride or LY294002 once daily (at 10:00 h). The animals were also intraperitoneally administered saline or LPS once daily (at 16:00 h) for 14 days. The doses of ASD, LPS, minocycline and LY294002 were chosen based on previous studies (Guo et al., 2019; Zhang et al., 2020c; Bassett et al., 2021; Silva et al., 2021).

### 2.5 Behavioral testing

#### 2.5.1 Sucrose preference test (SPT)

The SPT was performed as described (Zhang J. et al., 2021). Briefly, mice were individually housed, deprived of food and water for 12 h, and then given access to 1% sucrose solution (A) and water (B) for 2 h. The bottle positions were switched daily to avoid a side bias. The sucrose preference was calculated each week for each mouse using the formula: 100 × [VolA/(VolA + VolB)]. The sucrose consumption was normalized to body weight for each mouse.

#### 2.5.2 Forced swimming test (FST)

The FST was performed as described (Zhang J. et al., 2021). Briefly, at 24 h before the test, each mouse was individually placed in a glass cylinder (height: 25 cm, diameter: 15 cm) filled with 26 °C water to a depth of 15 cm for 15 min. The next day, the mice were placed once again in the same situation for 6 min. The entire process was recorded with a high-definition camera. An observer blinded to animal group recorded the time spent immobile during the last 4 min.

#### 2.5.3 Elevated plus maze test (EPMT)

The elevated plus maze contained two open arms (35 × 5 cm) and two closed arms (35 × 5 cm), connected by a central area (5 × 5 cm). The apparatus was lifted 50 cm above the floor. Tests were carried out in a quiet, dimly lit environment. The apparatus was wiped clean with 75% ethanol between tests. Mice were introduced to the maze at 1 h after the end of open field test. Mice were gently placed in the central area such that they faced one of the open arms. Spontaneous activity was monitored during 5 min. The number of entries into open arms as well as the total time spent in open arms were determined using EMP100 software (Taimeng Tech, Chengdu, China).

#### 2.5.4 Novel object recognition test (NORT)

The novel object recognition test was performed as described (Zhang et al., 2021). Mice were individually placed for 5 min in a Plexiglass arena measuring 40 × 60 cm with walls 30 cm high, and exploration was quantified using OFT100 video tracking software (Taimeng Tech, Chengdu, China). Subsequently, mice were subjected to three habituation sessions in which two objects identical in shape, color, and odor were introduced into the arena for 3 min at a 2-min interval between trials. Before the last session, one of the objects was replaced with a novel object. The time spent exploring each object was scored during each session.

#### 2.5.5 Morris water maze (MWM)

Spatial learning and memory were assessed in a Morris water maze. Experiments were carried out in a blue circular pool (diameter, 150 cm; height, 60 cm) filled to a depth of 30 cm with water rendered opaque by addition of 500 g milk powder and 30 ml blue food coloring. The area of the pool was divided into four virtual quadrants (NW, SW, SE, NE).

The experimental protocol consisted of four training days and an additional probe trial day. On training days, a platform was placed in the center of the SW quadrant, and the mice had to learn the location of the hidden platform with the help of visual cues placed around the maze. All animals had to perform four trials per training day. In each trial, the mice were placed into different quadrants (varied in a clockwise direction) and allowed to search for the hidden platform for 2 min. During the trials, the escape latency (time until the platform was found), the swimming path length to the platform and the swimming speed of the animals were measured and analyzed using Ethovision XT10 software (Noldus, Wageningen, Netherlands). If the platform was not found during the trial, rats were put on the platform for 10 s, and the escape latency was recorded as 2 min. The performance of the animals on the first training day was used to evaluate their short-term memory performance.

### 2.6 Primary culture of microglia and treatments

Primary microglia were isolated from brains of neonatal C57BL/6J mice (P0–P3) as described (Zhang et al., 2020b). After removing the meninges and blood vessels, brains were minced and centrifuged at 800 g for 5 min. The pellet was dissociated in 0.0125% trypsin (Gibco, CA, USA) for 10 min. The suspension was passed through a 70-μm cell strainer (Koch Membrane Systems, KS, USA). Cell pellets were harvested, washed, and cultured at 37°C in DMED–F12 medium containing 10% fetal bovine serum (FBS; Gibco). This procedure gave rise to mixed cultures in which microglia grew loosely atop a layer of tightly adhering astrocytes. Microglia were harvested by mechanical shaking, then transferred to new culture dishes. The purity of microglia was verified using immunofluorescence based on labeling with anti-Iba1 antibodies to identify microglia and with DAPI to identify nuclei. Cultures in which >98% of cells were Iba1+ were used in subsequent experiments.

### 2.7 Culture of adult NSPCs

Adult NSPCs were obtained from the subventricular zone of eight-week-old male C57BL/6J mice. The entire subventricular zone was dissected, and the lateral wall of the lateral ventricles was carefully removed from the surrounding brain tissue and collected in phosphate-buffered saline (PBS; BOSTER, Wuhan, China). These tissues were chopped into 1-mm cubes and digested in 0.125% trypsin (Gibco) for 5 min, then the digestion was stopped with soybean trypsin inhibitor. Cells were resuspended in complete DMEM/F12 medium (Gibco) containing 20 ng/ml recombinant murine fibroblast growth factor (FGF; PeproTech, NJ, USA), 20 ng/ml recombinant murine epidermal growth factor (EGF; PeproTech), 1% N-2 supplement (Gibco), and 2% B-27 supplement (Gibco). After culture for 7 days, neurospheres were isolated by centrifugation (600 g for 3 min), enzymatically dissociated into a single-cell suspension using 0.125% pancreatin (Sigma-Aldrich), and plated at a density of 5 × 104 cells/cm2 in proliferation medium. To permit serial cell passaging, this pancreatin dissociation process was repeated every 3–4 days.

### 2.8 Culture of NSPCs with conditioned medium from LPS-treated microglia

Microglia were plated at a density of 5 × 105 cells/cm2 and treated with either LPS or PBS for 24 h, washed twice with PBS, then cultured in DMEM-F12 + GlutaMax medium (Gibco) for another 24 h. The microglial medium was collected and used as conditioned medium to stimulate NSPC differentiation and proliferation. NSPCs were cultured in conditioned media from microglia treated with LPS (LPS-M-CM) or PBS (PBS-M-CM). These NSPCs were treated with 0, 10, 50 or 100 μM ASD, 50 ng/ml recombinant mouse brain-derived neurotrophic factor (BDNF; AmyJet, Wuhan, China) or 5 μM LY294002 (MedChemExpress). NSPC proliferation, differentiation, and survival were evaluated by immunofluorescence staining as described in sections 2.12.

### 2.9 Analysis of NSPC proliferation *in vitro* and *in vivo*

NSPCs were cultured in proliferation medium (97% DMEM/F12 + 20 ng/ml FGF +20 ng/ml EGF +1% N-2 supplement +2% B-27 supplement) and treated with different concentrations of ASD (10, 50, or 100 μM) or 50 ng/ml BDNF in the presence of PBS-M-CM or LPS-M-CM. The number and size of neurospheres were assessed after 4 days of culture.

To examine NSPC proliferation in the brain, mice received intraperitoneal injections of 5′-bromo-2′deoxyuridine (BrdU; Sigma-Aldrich) at a daily dose of 50 mg/kg for 3 days. Mice were euthanized at 24 h after the last injection, and proliferating cells in the hippocampus were labeled with anti-BrdU antibody as described below. The number of BrdU + cells was quantified.

### 2.10 Analysis of NSPC differentiation *in vitro* and *in vivo*

NSPCs were cultured in differentiation medium (93% DMEM/F12 + 1% N-2 supplement +2% B-27 supplement +5% FBS) and treated with different concentrations of ASD (10, 50, or 100 μM) or 50 ng/ml BDNF in the presence of PBS-M-CM or LPS-M-CM. After 5 days in culture, the rates of NSPC differentiation into neurons or astrocytes were determined by quantifying the percentages of DCX + or GFAP + or NG2+ cells as described in section 2.12.

To examine NSPC differentiation in the brain, mice received intraperitoneal injections of BrdU at a daily dose of 50 mg/kg for 3 days. Mice were euthanized at 7 days after the last injection. Using immunocytochemistry of hippocampal sections as described below, we identified neurons that differentiated from progenitor cells based on double labeling with anti-BrdU and anti-DCX antibodies, and we identified astrocytes that differentiated from progenitor cells based on double labeling with anti-BrdU and anti-GFAP antibodies. Differentiation of hippocampal NSPCs was assessed by quantifying the number and percentage of BrdU + -DCX + cells or BrdU + -GFAP + cells.

### 2.11 Analysis of NSPC survival and newborn neuron maturation *in vitro* and *in vivo*

NSPCs were cultured in proliferation medium for 24 h, then treated with BrdU for another 12 h. The culture medium was replaced with PBS-M-CM or LPS-M-C, to which were added different concentrations of ASD (10, 50, or 100 μM) or 50 ng/ml BDNF. After 7 days in differentiation culture, the rate of NSPC survival was determined by quantifying the percentage of BrdU + cells as described below.

To evaluate NSPC survival and newborn neuron maturation in the brain, mice received intraperitoneal injections of BrdU at a daily dose of 50 mg/kg for 3 days, after which animals were administered LPS, ASD, minocycline or LY294002 as described in sections 2.4. Mice were euthanized at 14 days after the last injection. The rate of NSPC survival was determined by quantifying the number of BrdU + cells as described below. Mature neurons derived from progenitor cells were identified based on double labeling with anti-BrdU and anti-NeuN antibodies.

### 2.12 Immunocytochemistry

Brain tissue was prepared and stained as described (Zhang et al., 2020b). Differentiated NSPCs were plated at a density of 105 cells/cm2 and fixed with 4% paraformaldehyde (pH 7.2) for 30 min. Hippocampal tissue or primary cells in culture were permeabilized with 0.5% Triton X-100 in PBS for 15 min, blocked in 10% donkey serum for 2 h, then incubated overnight at 4 °C with the following primary antibodies: goat anti-Iba1 (1:400, Abcam, Cambridge, United Kingdom), goat anti-Doublecortin (DCX; 1:400, Santa Cruz Biotechnology, CA, USA), mouse anti-BrdU (1:400, Cell Signaling Technology, MA, USA), and rabbit anti-GFAP (1:400, Cell Signaling Technology).

Tissue or cells were then incubated for 2 h at room temperature with DyLight 549-conjugated donkey anti-goat or DyLight 488-conjugated donkey anti-mouse secondary antibodies (both 1:300, Jackson ImmunoResearch, PA, USA). Finally, cells were incubated with DAPI (1:10,000, Roche, Basel, Switzerland) for 5 min and imaged using a fluorescence microscope (IX73, Olympus, Tokyo, Japan). Cell numbers were quantified using GraphPad Prism 5.0 (version 8.0, SPSS Inc., Chicago, USA).

### 2.13 Enzyme-linked immunosorbent assay (ELISA)

The dentate gyrus was dissociated from slices containing the hippocampus, flash-frozen in liquid nitrogen, and homogenized. Samples were centrifuged at 1, 000 g for 30 min. The concentration of total protein was determined using the BCA kit (BOSTER, Wuhan, China). After each sample was diluted to 1 g/ml, supernatants were assayed for interleukin (IL)-1β using an ELISA kit (BOSTER) according to the manufacturer’s protocols, and IL-1β concentration per g of total protein was calculated. The manufacturer-specified detection limit was 4 pg/ml.

### 2.14 Western blotting

Western blotting was used to assess levels of phosphatidylinositol 3 kinase/serine-threonine kinase (PI3K/Akt) signaling in hippocampus and in NSPCs. Mice were perfused with PBS, hippocampi were isolated and homogenized. Hippocampi or primary cultures of NSPCs were sonicated in RIPA buffer containing protease and phosphorylase kinase inhibitors (Solarbio, Beijing, China). Protein lysate was centrifuged at 1,000 g for 30 min, then the supernatant was fractionated on 12% Tris-glycine SDS-PAGE gels, transferred to PVDF membranes (0.2 or 0.45 μm), and incubated with antibodies against PI3K (1:600, Cell Signaling Technology), AKT (1:400, Abcam), or phospho-AKT (pAKT; 1:600, Abcam). Membranes were incubated with the primary antibody overnight at 4 °C, washed with PBS (BOSTER), then incubated with secondary antibodies (1:10,000, Abcam) for 2 h at room temperature. Fluorescence was developed using the ECL-Plus kit (Millipore, MA, USA) and quantified using ImageJ software (version 1.45J; National Institutes of Health, Bethesda, MD, USA).

### 2.15 Statistical analysis

GraphPad Prism software (version 6.0; GraphPad Software, Chicago, USA) was used for all statistical analyses. Data were presented as the mean ± standard error of the mean (SEM). Pairwise comparisons were assessed for significance using Student’s two-tailed *t* test, and comparisons of three or more values were assessed using one- or two-way ANOVA and Tukey’s multiple-comparisons test. Differences were considered statistically significant if *p* < 0.05.

## 3 Results

### 3.1 ASD protects NSPCs from the microglia-mediated inflammatory niche, promoting their proliferation, survival and neuronal differentiation *in vitro* by activating the PI3K-Akt pathway

**FIGURE 1:**
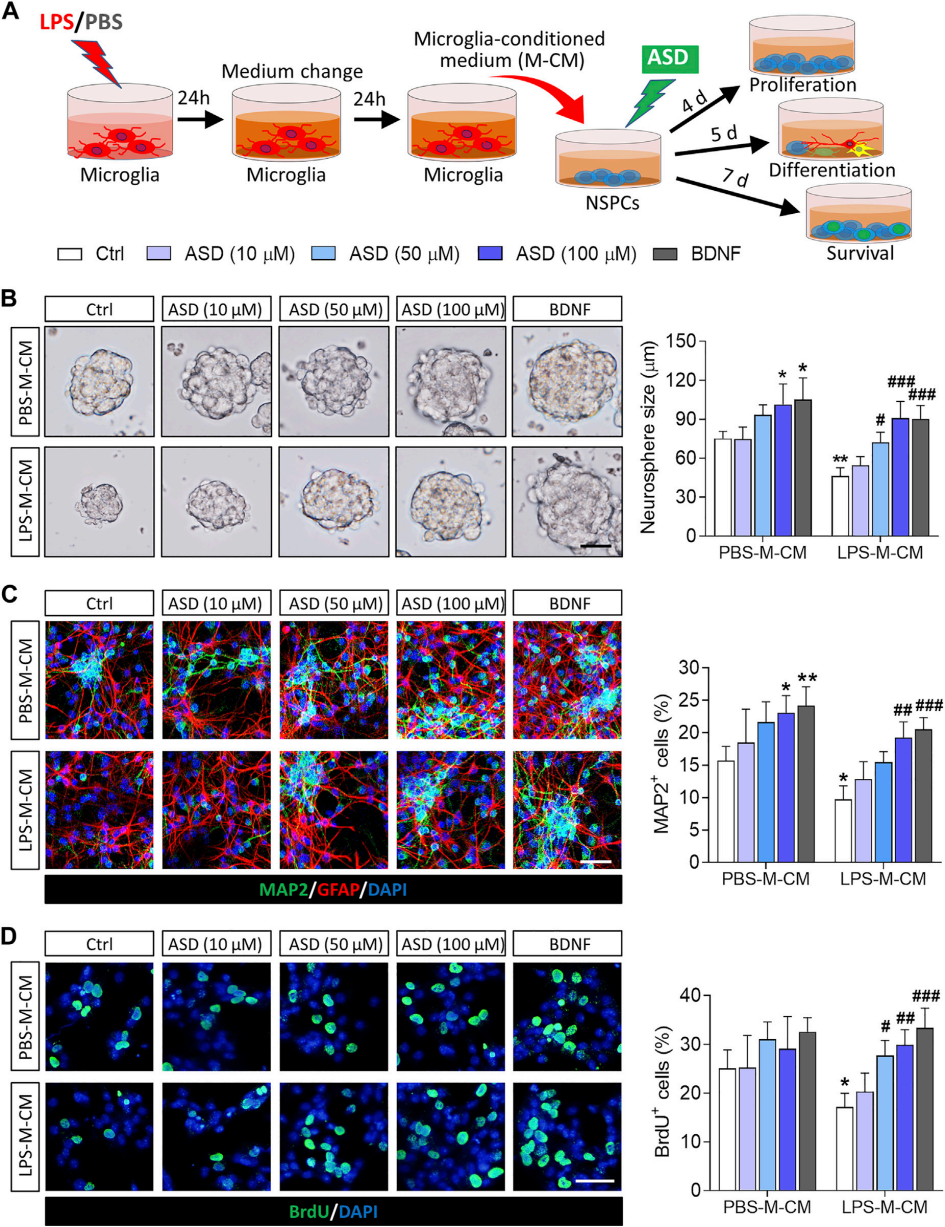
Effects of ASD on NSPC proliferation, survival and neuronal differentiation in the presence or absence of a microglia-mediated inflammatory niche. **(A)**, Scheme describing the experimental evaluation of the effects of akebia saponin D (ASD) on survival of neurospheres in the presence or absence of an inflammatory niche. Microglia were treated with phosphate-buffered saline (PBS) or lipopolysaccharide (LPS) for 24 h, and the microglia-conditioned medium (M-CM) was collected. Neural stem/precursor cells (NSPCs) were treated with different concentrations of ASD (0, 10, 50, or 100 μM) in the presence of PBS-M-CM or LPS-M-CM. Brain-derived neurotrophic factor (BDNF) at 50 ng/ml was used as control. NSPC proliferation was measured after 4 days in culture; differentiation, after 5 days; and survival, after 7 days. **(B)**, Micrographs and quantification of neurosphere size under different treatment conditions. Scale bar, 100 μm. **(C)**, Micrographs and quantification of pleiotropic NSPC differentiation under different treatment conditions. Astrocytes were labeled with antibody against GFAP (green); neurons, with antibody against microtubule-associated protein 2 (MAP2) (red). Scale bar, 50 μm. **(D)**, Micrographs and quantification of NSPC survival under different treatment conditions. Surviving NSPCs were labeled with BrdU (green). Scale bar, 20 μm. Results for each group were averaged from 5 micrographs (40×) from each of 4-6 slides. Data are mean ± standard error of the mean (SEM). *p < 0.05, **p < 0.01 vs. control microglia conditioned medium (PBS-M-CM), #*p* < 0.05, ##*p* < 0.01, ###*p* < 0.001 vs. LPS-treated microglia conditioned medium (LPS-M-CM) by two-way ANOVA with Tukey’s multiple-comparisons test.

To confirm that ASD directly regulates NSPCs, we examined the effects of ASD on NSPC proliferation, survival and neuronal differentiation *in vitro*. To simulate the microglia-mediated inflammatory niche, primary cells or neurospheres were cultured in conditioned medium from primary microglial cultures (Figure 1A). Like BDNF, 100 μM ASD increased the diameter of neurospheres in PBS-M-CM. LPS-M-CM decreased the diameter of the neurospheres, which ASD reversed at concentrations of 50 or 100 μM, but not 10 μM (Figure 1B).

To confirm that ASD promotes NSPC differentiation, NSPCs were allowed to differentiate for 7 days in differentiation medium in the presence or absence of ASD. Then, we analyzed the percentages of cells that were developmentally committed to becoming neurons (DCX + cells) or astrocytes (GFAP + cells). LPS-M-CM reduced the neuronal differentiation of NSPCs, while either BDNF or ASD (50 or 100 μM) promoted it in the presence or absence of LPS-M-CM (Figure 1C).

To confirm that ASD promotes NSPC survival, we used BrdU to label proliferating NSPCs, then these cells were allowed to differentiate for 7 days in differentiation medium. Exposing NSPCs to LPS-M-CM decreased the percentage of BrdU + cells, which 50 or 100 μM ASD partially reversed (Figure 1D).

**FIGURE 2:**
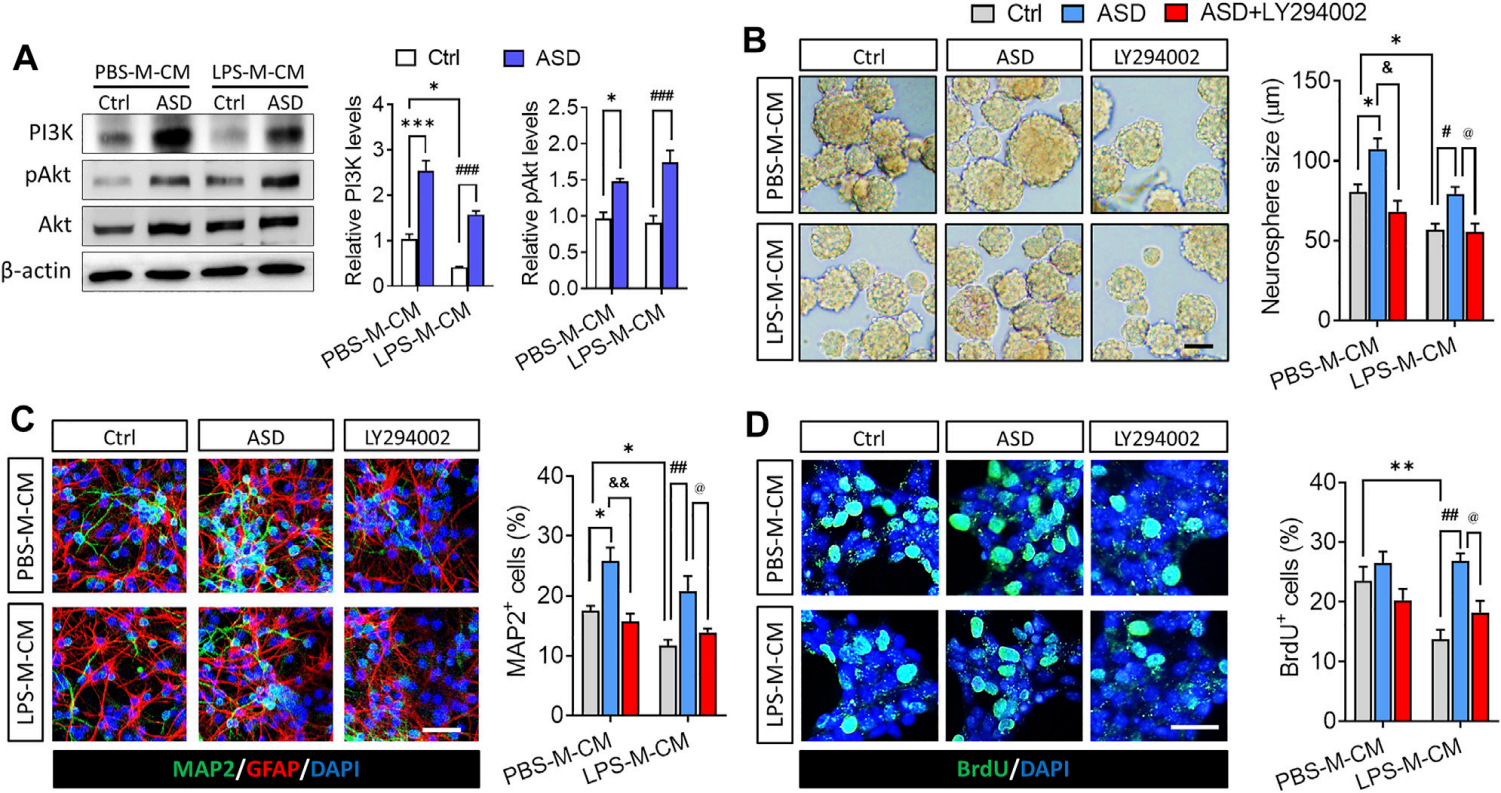
ASD protects NSPCs from the microglia-mediated inflammatory niche by activating the PI3K-AKT pathway. **(A)**, Western blotting showing activation of the PI3K-AKT pathway in NSPCs after ASD treatment (100 μM) in the presence of PBS-M-CM or LPS-M-CM. Levels of PI3K and AKT were normalized to those of β-actin, and levels of phospho-AKT (p-AKT) were normalized to those of AKT. Ctrl, control without ASD. **(B)**, Micrographs and quantification of neurosphere size, showing that the PI3K-AKT pathway inhibitor LY294002 blocked the effects of ASD on neurosphere proliferation in the presence of PBS-M-CM or LPS-M-CM. Scale bar, 100 μm. **(C)**, Micrographs and quantification of the percentage of MAP2+ cells, showing that LY294002 also blocked the effects of ASD on neurogenesis in the presence of PBS-M-CM or LPS-M-CM. Astrocytes were labeled with antibody against GFAP (green); neurons, with antibody against MAP2 (red). Scale bar, 50 μm. **(D)**, Micrographs and quantification of the percentage of BrdU + cells, showing that LY294002 blocked the effects of ASD on NSPC survival in the presence of LPS-M-CM. Surviving NSPCs were labeled with BrdU (green). Scale bar, 20 μm. Results for each group were averaged from 5 micrographs (40×) from each of 4-6 slides. Data are mean ± standard error of the mean (SEM), *p < 0.05, **p < 0.01, ***p < 0.001 vs. control microglia conditioned medium (PBS-M-CM), #*p* < 0.05, ##*p* < 0.01, ###*p* < 0.001 vs. LPS-treated microglia conditioned medium (LPS-M-CM), &*p* < 0.05, &&*p* < 0.01 vs. ASD + PBS-treated microglia conditioned medium (ASD + PBS-M-CM), @*p* < 0.05 vs. ASD + LPS-treated microglia conditioned medium (ASD + LPS-M-CM) by two-way ANOVA with Tukey’s multiple-comparisons test.

Next, we confirmed in this *in vitro* system that the PI3K-Akt pathway mediates the effects of ASD on NSPC proliferation, survival and neuronal differentiation. Conditioned medium from microglia treated only with LPS reduced levels of PI3K, Akt, and pAkt in primary cultures of NSPCs, while ASD (100 μM) strongly increased those levels in the presence or absence of LPS-M-CM (Figure 2A). In addition, the PI3K-Akt pathway inhibitor LY294002 blocked the effects of ASD on NSPC proliferation and neuronal differentiation in the presence or absence of LPS-M-CM (Figures 2B–D).

These results suggest that ASD protects NSPCs from the microglia-mediated inflammatory niche and promotes their proliferation and neurogenesis by activating the PI3K-Akt pathway.

### 3.2 ASD protects hippocampal neurogenesis from the chronic LPS-induced inflammatory niche *in vivo* by activating the PI3K-Akt pathway

**FIGURE 3:**
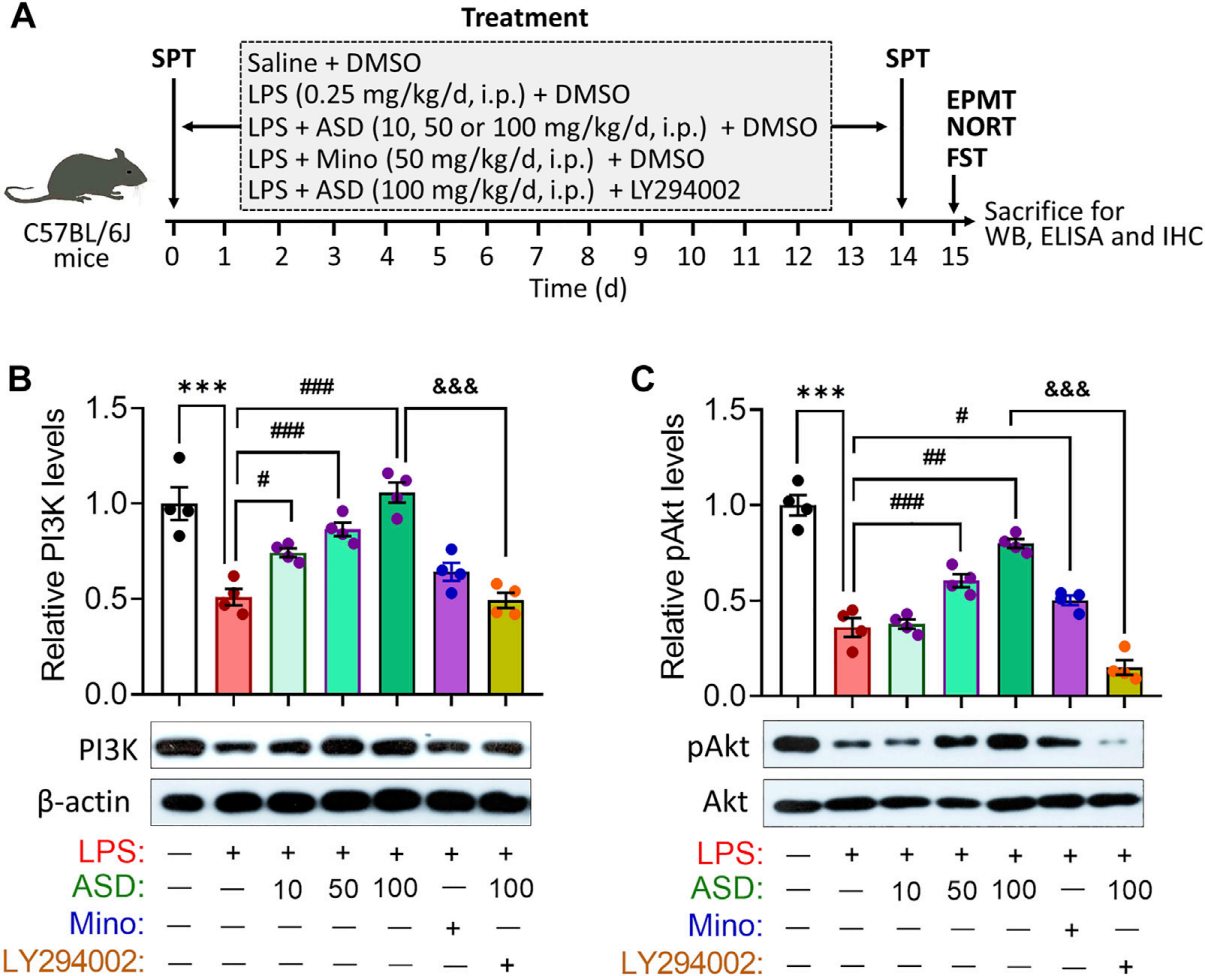
ASD activates the PI3K-Akt pathway in hippocampus of mice chronically exposed to LPS. **(A)**, Scheme of the experimental procedure. ASD, akebia saponin D; DMSO, dimethyl sulphoxide; ELISA, enzyme-linked immunosorbent assay; EPMT, elevated plus maze test; FST, forced swimming test; IHC, immunocytochemistry; LPS, lipopolysaccharide; Mino, minocycline; NORT, novel object recognition test; SPT, sucrose preference test; WB, western blotting. **(B,C)**, Western blotting shows the levels of PI3K and pAkt in the hippocampus of mice treated with saline (Ctrl) or lipopolysaccharide (LPS), then with akebia saponin D (ASD), minocycline (Mino) or PI3K-Akt inhibitor (LY294002). Levels of PI3K were normalized to those of β-actin, and levels of pAkt were normalized to those of Akt. Data are mean ± standard error of the mean (SEM) (n = 4), ***p < 0.001 vs. Ctrl group, #*p* < 0.05, ##*p* < 0.01, ###*p* < 0.001 vs. LPS group, &&&*p* < 0.001 vs. ASD (100 mg/kg) + LPS group based on one-way ANOVA with Tukey’s multiple-comparisons test.

Given the neuroprotective effects of ASD, we evaluated its effects on hippocampal neurogenesis and behavior in a mouse model of chronic neuroinflammation, induced by chronic exposure to LPS (Figure 3A). Given the role of PI3K-Akt signaling in regulation of stem cell function and improvement of cognitive deficits (Yu et al., 2012; Ke et al., 2016), we first examined the effects of ASD on PI3K-Akt signaling in the hippocampus. We found that chronic LPS exposure caused a striking reduction in the levels of PI3K and pAkt in hippocampus, which ASD reversed in a dose-dependent manner (Figures 3B,C).

**FIGURE 4:**
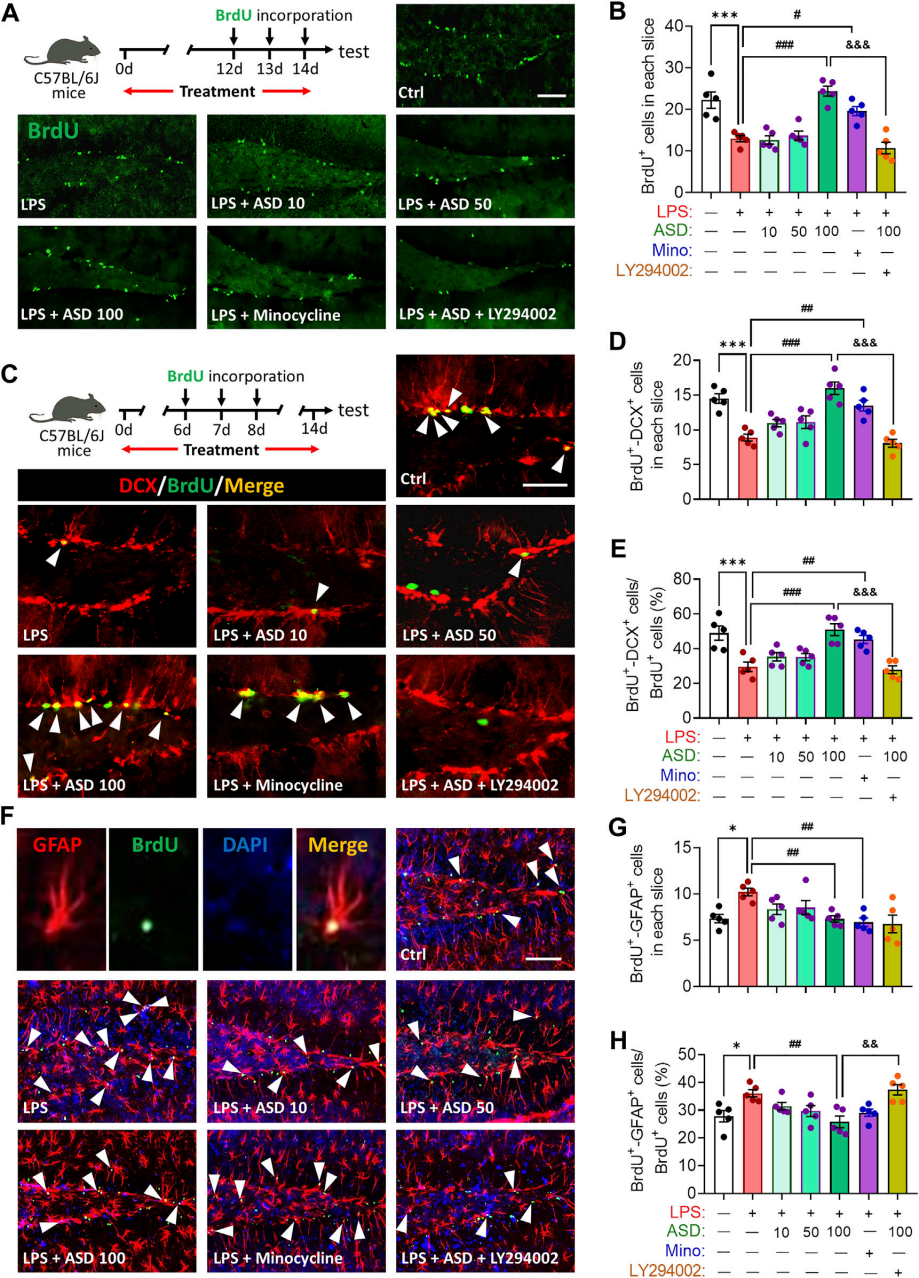
Effects of ASD on NSPC proliferation and differentiation in dentate gyrus of mice chronically exposed to LPS. **(A)**, Timeline for detecting proliferation of neural stem/progenitor cells (NSPCs) based on immunofluorescence micrographs of BrdU + cells in dentate gyrus (DG) of mice. Proliferating NSPCs were labeled using 5′-bromo-2′deoxyuridine (BrdU) (green). Scale bar, 100 μm. **(B)**, Quantification of hippocampal BrdU + cells. Five mice from each group were examined, and five hippocampal micrographs (40×) from each animal were quantified. **(C)**, Timeline for evaluating NSPC differentiation based on immunofluorescence micrographs of BrdU + -DCX + cells in dentate gyrus (DG) of mice. Proliferating NSPCs were labelled with BrdU (green); immature neurons, with antibody against doublecortin (DCX); and newborn neurons differentiated from NSPCs, with both BrdU and anti-DCX antibody (white arrowheads). Scale bar, 100 μm. **(D)**, Quantification of the hippocampal BrdU + -DCX + cells in each slice. **(E)**, Quantification of the percentage of total BrdU + cells in the DG that were BrdU + -DCX+. **(F)**, Immunofluorescence micrographs of BrdU + -GFAP + cells in the DG. Proliferating NSPCs were labelled with BrdU (green); astrocytes, with antibody against GFAP; and newborn astrocytes differentiated from NSPCs, with BrdU and anti-GFAP antibody (white arrowheads). Scale bar, 100 μm. **(G)**, Quantification of hippocampal BrdU + -GFAP + cells in each slice. **(H)**, Quantification of the percentage of total BrdU + cells in the DG that were BrdU + -GFAP+. Five mice from each group were examined, and five hippocampal micrographs (40×) from each animal were quantified. Each dot in the bar graph represents the average of all micrographs for each mouse. Data are mean ± standard error of the mean (SEM) (n = 5), *p < 0.05, ***p < 0.001 vs. Ctrl group, #*p* < 0.05, ##*p* < 0.01, ###*p* < 0.001 vs. LPS group, &&*p* < 0.01, &&&*p* < 0.001 vs. ASD (100 mg/kg) + LPS group by one-way ANOVA with Tukey’s multiple-comparisons test. Each dot in the bar graph represents the average of all micrographs for each mouse.

**FIGURE 5:**
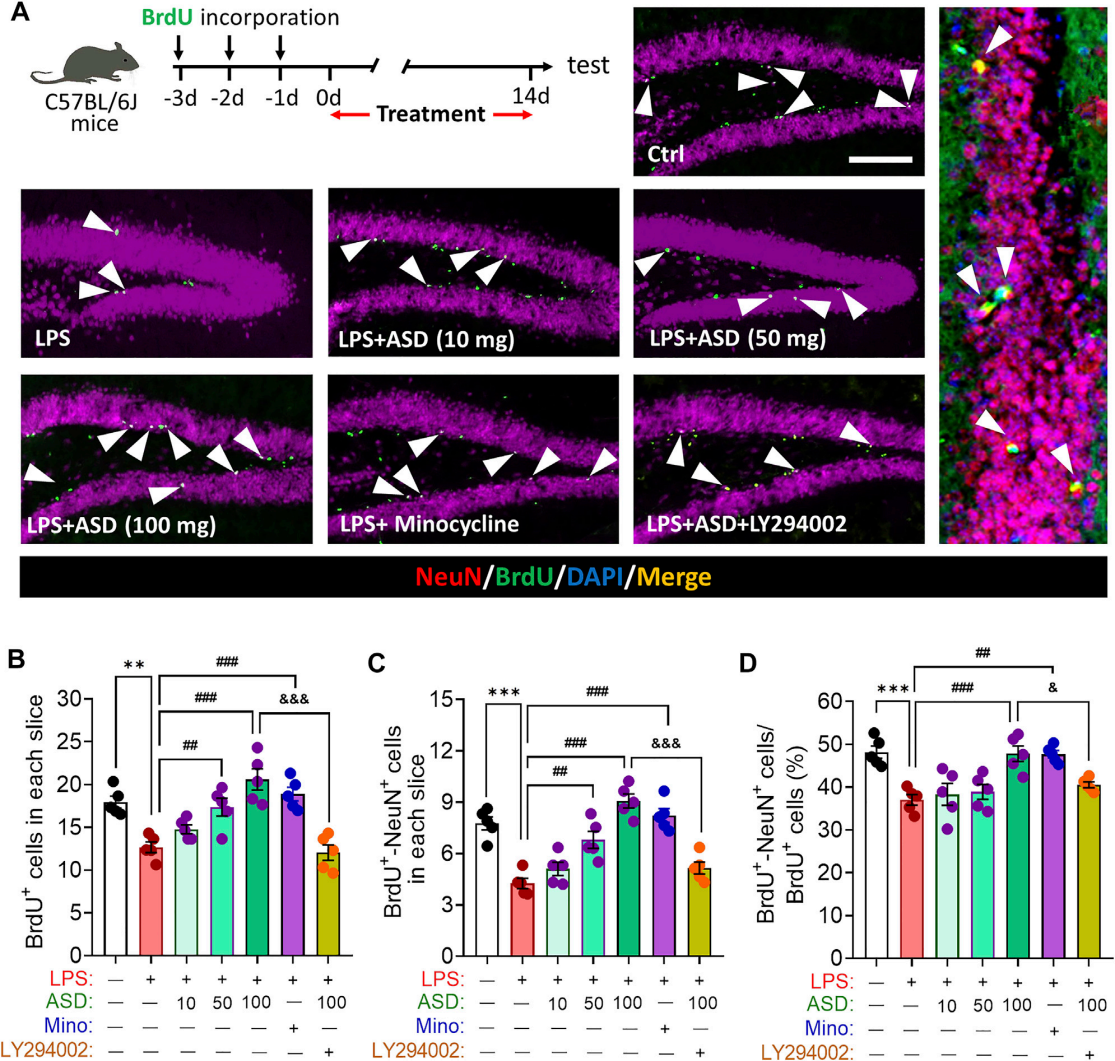
Effects of ASD on survival and maturation of newborn neurons in dentate gyrus of mice chronically exposed to LPS. **(A)**, Timeline for evaluating newborn neuron survival and maturation based on immunofluorescence micrographs of BrdU + -NeuN + cells in the dentate gyrus of mice. Surviving cells were labelled with BrdU (green); mature neurons, with antibody against neuron-specific nucleoprotein (NeuN); and mature neurons differentiated from NSPCs, with both BrdU and anti-NeuN antibody (white arrowheads). Scale bar, 100 μm. **(B)**, Quantification of hippocampal BrdU + cells in each slice. **(C)**, Quantification of hippocampal BrdU + -NeuN + cells in each slice. **(D)**, Quantification of the percentage of total BrdU + cells in DG that were BrdU + -NeuN+. Five mice from each group were examined, and five hippocampal micrographs (40×) from each animal were quantified. Each dot in the bar graph represents the average of all micrographs for each mouse. Data are mean ± standard error of the mean (SEM) (n = 5), **p < 0.01, ***p < 0.001 vs. Ctrl group, ##*p* < 0.01, ###*p* < 0.001 vs. LPS group, &*p* < 0.05, &&&*p* < 0.001 vs. ASD (100 mg/kg) + LPS group by one-way ANOVA with Tukey’s multiple-comparisons test.

LPS-induced microglial hyperactivation should supress neurogenesis (Littlefield et al., 2015; Bassett et al., 2021), which we confirmed by showing that LPS strongly reduced the numbers of BrdU + cells (proliferating cells) (Figures 4A,B), BrdU + -DCX + cells (newborn neurons) (Figures 4C–E) and BrdU + -NeuN + cells (mature neurons) (Figures 5A–D) in hippocampus, while increasing the number of GFAP + -BrdU + cells in the SGZ of the hippocampus (Figures 4F–H). Like minocycline, ASD at 100 mg/kg/d for 14 days strongly reversed these LPS effects, while the effects of ASD were blocked by the PI3K-Akt signaling inhibitor LY294002 (Figures 4A–H, 5A–D). We also found that ASD at 100 mg/kg/d for 14 days promoted the proliferation of neural stem cells in normal mice, but did not affect hippocampal neurogenesis and the maturation of newborn neurons (Supplementary Figure S1).

**FIGURE 6:**
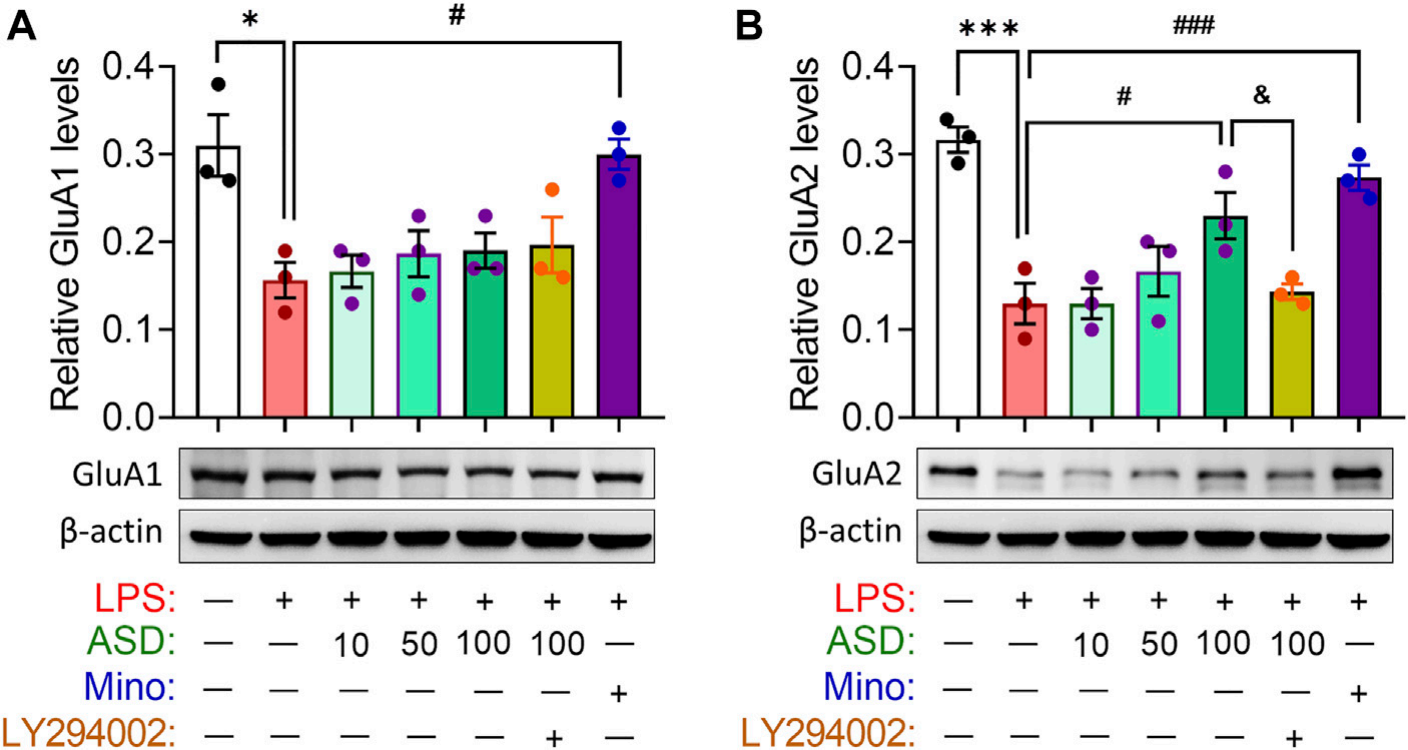
Effects of ASD on synaptic function in hippocampus of mice chronically exposed to LPS. **(A,B)**, Western blotting shows the levels of GluA1 and GluA2 in the hippocampus of mice treated with saline (Ctrl) or lipopolysaccharide (LPS), then with akebia saponin D (ASD), minocycline (Mino) or PI3K-Akt inhibitor (LY294002). Levels of GluA1 and GluA2 were normalized to those of β-actin. Figures 6A,B share the same β-actin. Data are mean ± standard error of the mean (SEM) (n = 4), *p < 0.05, ***p < 0.001 vs. Ctrl group, #*p* < 0.05, ###*p* < 0.001 vs. LPS group, &*p* < 0.05 vs. ASD (100 mg/kg) + LPS group based on one-way ANOVA with Tukey’s multiple-comparisons test.

Abnormal neurogenesis in hippocampus will lead to synaptic dysfunction which link to depression- and anxiety-related symptoms aand cognitive function. Indeed, our results showed that chronic LPS exposure decreased the hippocampal levels of glutamate receptor 1 (GluA1) and 2 (GluA2) subunits of the *a*-amino-3-hydroxy-5-methyl-4-isoxazole-propionicacid (AMPA), which mediated rapid excitatory synaptic transmission in the central nervous system. These changes in synaptic function-related markers were partially reversed by ASD, while the effects of ASD were blocked by the PI3K-Akt signaling inhibitor LY294002 (Figures 6A,B).

**FIGURE 7:**
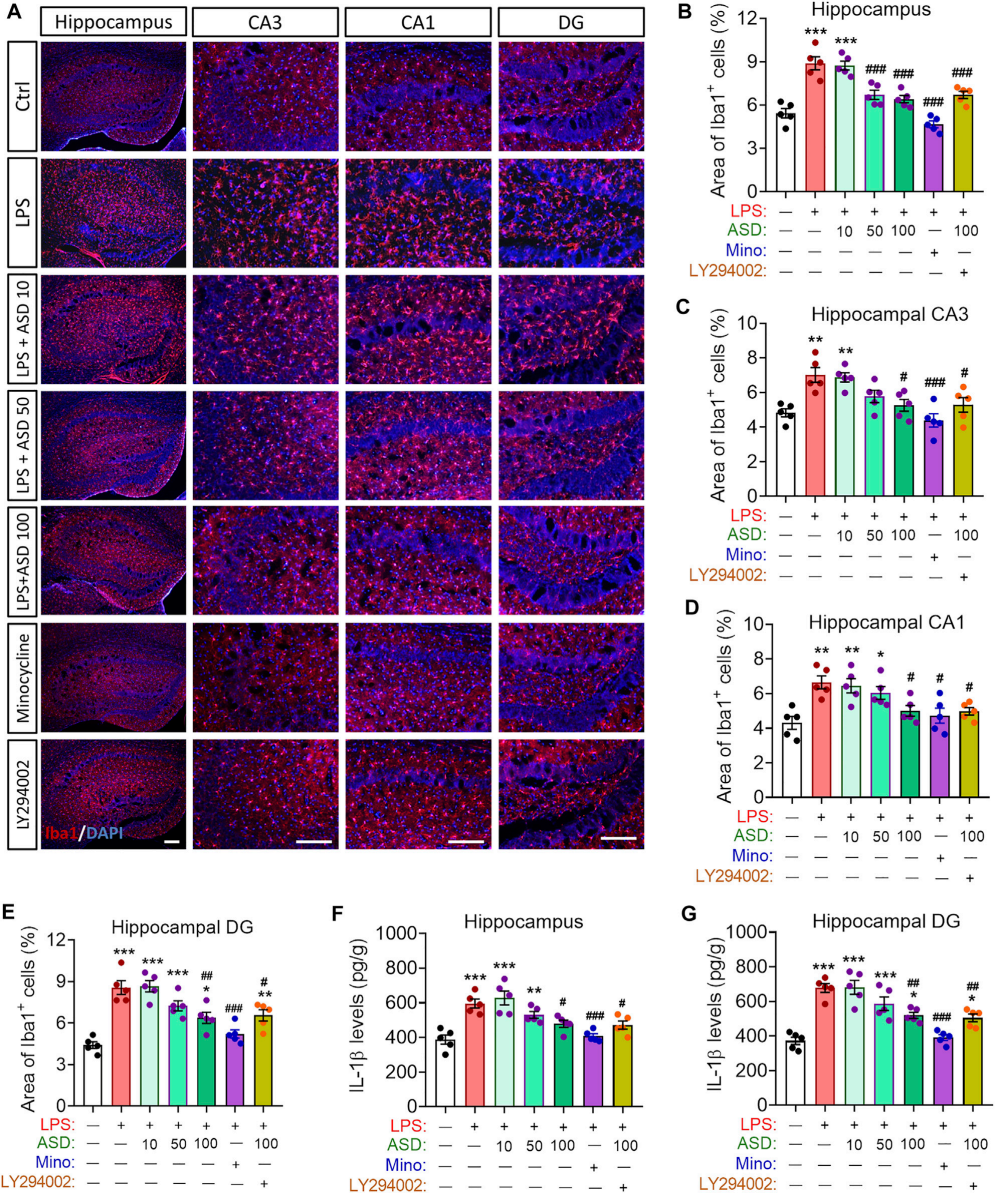
Effects of ASD on microglia, astrocyte numbers and IL-1β levels in neurogenic niche of mice chronically exposed to LPS. **(A)**, Representative fluorescence micrographs showing the morphology and density of microglia in the hippocampus of mice treated with saline (Ctrl) or lipopolysaccharide (LPS), followed by akebia saponin D (ASD) or minocycline. Microglia were labeled with antibody against ionized calcium binding adapter molecule 1 (Iba1) (red) and nuclei, with 4’,6-diamidino-2-phenylindole (DAPI) (blue). Scale bar, 100 μm. **(B–E)**, Quantification of the percentages of total area containing Iba1+ cells in hippocampal CA3 (C), CA1 (D) and dentate gyrus (DG) (E) for evaluating changes in the morphology and density of microglia. Five mice from each group were examined, and five hippocampal micrographs (40×) from each animal were quantified. Each dot in the bar graph represents the average of all micrographs for each mouse. **(F,G)**, Quantification of the concentration of IL-1β in hippocampus and hippocampal DG as an index of neuroinflammation. Data are mean ± standard error of the mean (SEM) (n = 5), *p < 0.05, **p < 0.01, ***p < 0.001 vs. Ctrl group, #*p* < 0.05, ##*p* < 0.01, ###*p* < 0.001 vs. LPS group by one-way ANOVA with Tukey’s multiple-comparisons test.

Given the observed improvement in hippocampal neurogenesis of LPS-administrated mice that were treated with ASD, we determined whether ASD inhibits microglial activation or acts directly on neural stem cells in neurogenic niche of mice exposed LPS stimulation. We found that LPS induced increases in the area of Iba1+ cells in hippocampus and in the level of IL-1β in hippocampus or hippocampal CA1, CA3 and dentate gyrus (Figures 7A–G), reflecting proliferation of microglia and their activation to secrete pro-inflammatory cytokines. Minocycline and ASD at 100 mg, but not 10 mg or 50 mg, reversed these effects of LPS in hippocampal CA1, CA3 (Figures 7A–G). However, even the 100 mg of ASD did not completely reverse the LPS-induce changes in morphology and number of microglia and the increase in IL-1β in hippocampal dentate gyrus (Figures 7A–G). These results suggest that ASD maybe directly activate PI3K-Akt signaling of hippocampal NSPC to promote neurognegesis rather than inhibition of microglial activation in dentate gyrus of LPS-exposed mice.

These experiments indicate that 100 mg/kg/d ASD activated the PI3K-Akt pathway to restore hippocampal NSPC proliferation, survival and neuronal differentiation, as well as the synaptic function of hippocampal neurons in a microenvironment of chronic neuroinflammation.

### 3.3 ASD ameliorates depressive- and anxiety-like behaviors and cognitive impairment in a mouse model of chronic neuroinflammation

In healthy individuals, a steady stream of new neurons in the DG can project into the cerebral cortex to regulate emotional and cognitive functions (Zhang et al., 2021), but this steady stream is likely compromised in patients with Alzheimer’s disease or depression, who suffer hippocampal atrophy. The impaired neurogenesis likely contributes to depression, anxiety and cognitive impairments. Given our observations above that ASD can promote NSPC proliferation and neuronal differentiation *in vitro* and *in vivo*, even in a chronically inflammatory microenvironment, we wanted to examine whether these effects of ASD could mitigate depression, anxiety and cognitive impairment due to chronic inflammation.

**FIGURE 8:**
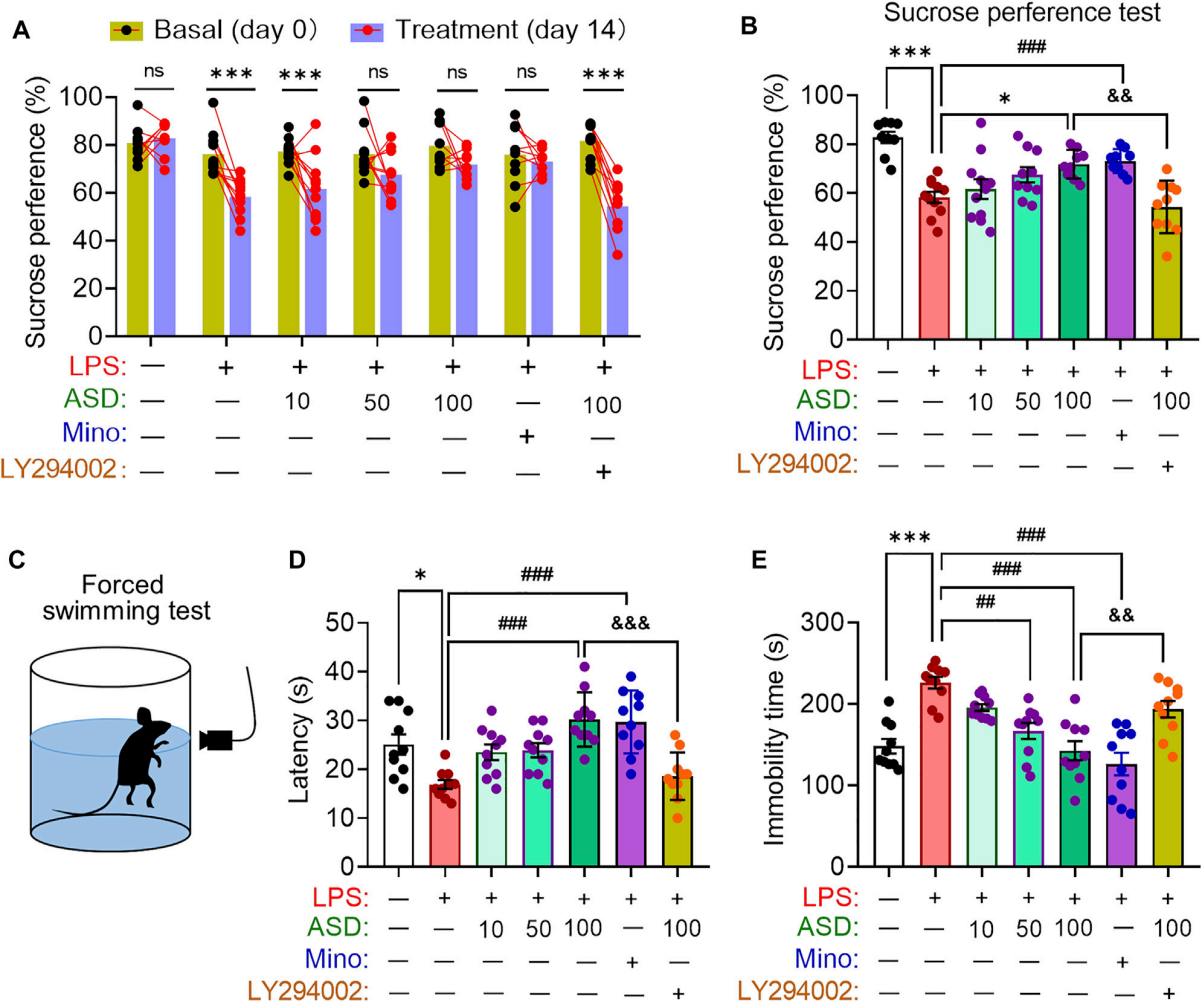
Effects of ASD on depressive-like behaviors of mice chronically exposed to LPS in the presence or absence of PI3K-Akt inhibitor. **(A)**, Changes in sucrose preference of individual saline-treated (Ctrl) or lipopolysaccharide (LPS)-treated mice, before treatment (day 0) and afterward (day 14). **(B)**, Changes in sucrose preference following treatment with akebia saponin D (ASD), minocycline (Mino) or PI3K-Akt inhibitor (LY294002) for 14 days. **(C–E)**, Effects of ASD on immobility time and latency in the forced swimming test. Data are mean ± standard error of the mean (SEM) (n = 8-11). Panel (A): ***p < 0.001 vs. basal (0-days) by a paired Student’s t test for. Panels (B), (D) and (E): *p < 0.05, ***p < 0.001 vs. Ctrl group, ##*p* < 0.01, ###*p* < 0.001 vs. LPS group, &&*p* < 0.01, &&&*p* < 0.001 vs. ASD (100 mg/kg) + LPS group by one-way ANOVA with Tukey’s multiple-comparisons test.

Depressive-like behaviors of mice were evaluated using the SPT, which evaluates anhedonia, and the FST, which assesses behavioral despair. Chronic exposure to LPS decreased the sucrose preference of mice, which ASD (at a daily dose of at least 100 mg/kg) or minocyline reversed (Figures 8A,B). Chronic exposure to LPS shortened the latency time and prolonged immobility time in the FST, which ASD reversed (Figures 8C–E).

**FIGURE 9:**
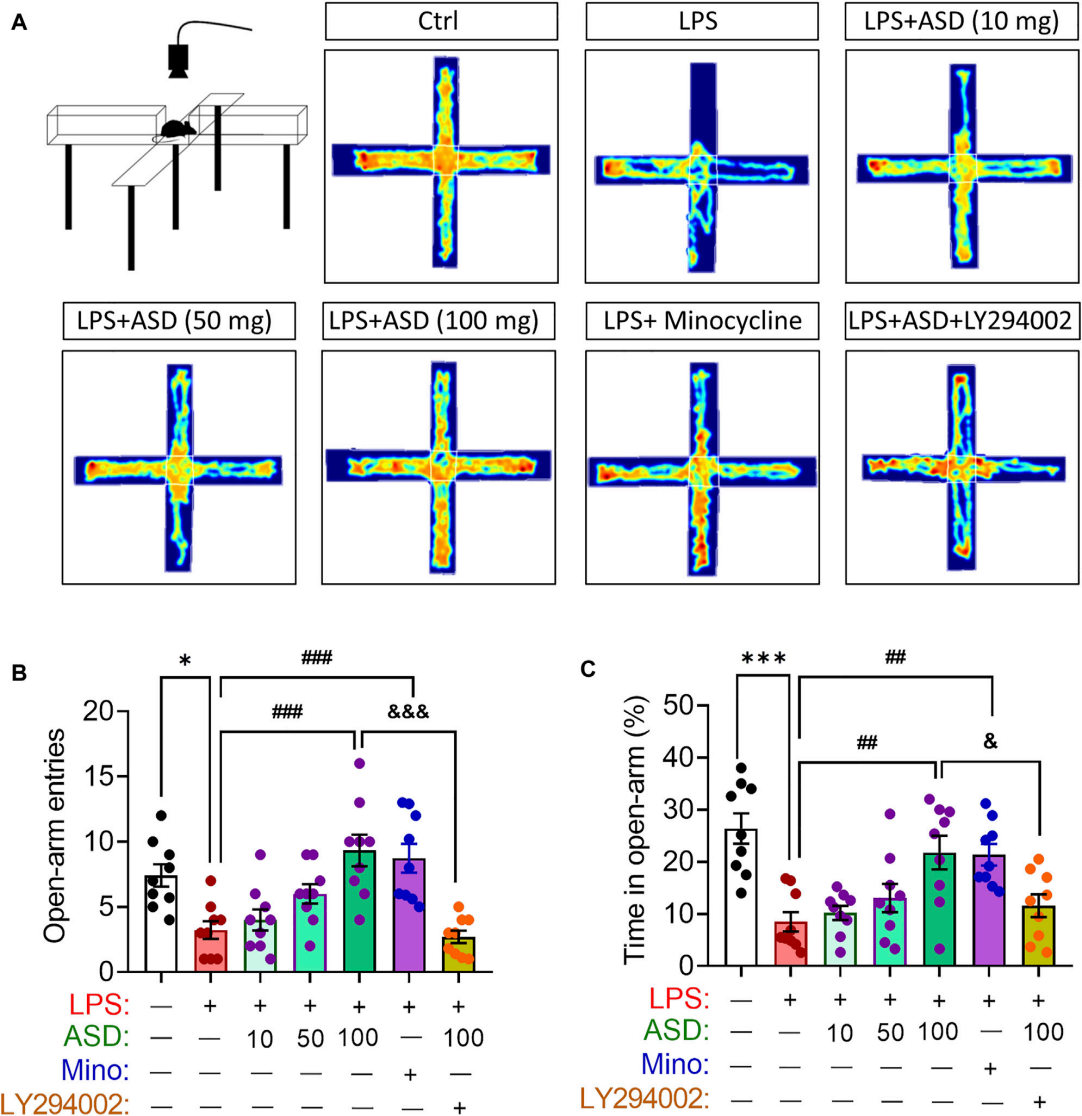
Effects of ASD on anxiety-like behaviors of mice chronically exposed to LPS in the presence or absence of PI3K-Akt inhibitor. **(A)**, Scheme describing the experimental evaluation of anxiety-like behaviors of mice using the elevated plus maze test, and the heatmap of mouse behavior in this test. **(B,C)**, Changes in open-arm entries and time in open-arms in mice treated with saline (Ctrl) or lipopolysaccharide (LPS), followed by akebia saponin D (ASD), minocycline (Mino) or PI3K-Akt inhibitor (LY294002) for 14 days. Data are mean ± standard error of the mean (SEM) (n = 8-11). *p < 0.05, ***p < 0.001 vs. Ctrl group, ##*p* < 0.01, ###*p* < 0.001 vs. LPS group, &*p* < 0.05, &&&*p* < 0.001 vs. ASD (100 mg/kg) + LPS group by one-way ANOVA with Tukey’s multiple-comparisons test.

Anxiety-like behaviors of mice were evaluated using the elevated plus maze test (Figure 9A). Chronic exposure to LPS reduced the number of open-arm entries and time spent in open arms, which ASD (at a daily dose of at least 100 mg/kg) or minocyline reversed (Figures 9B,C).

**FIGURE 10:**
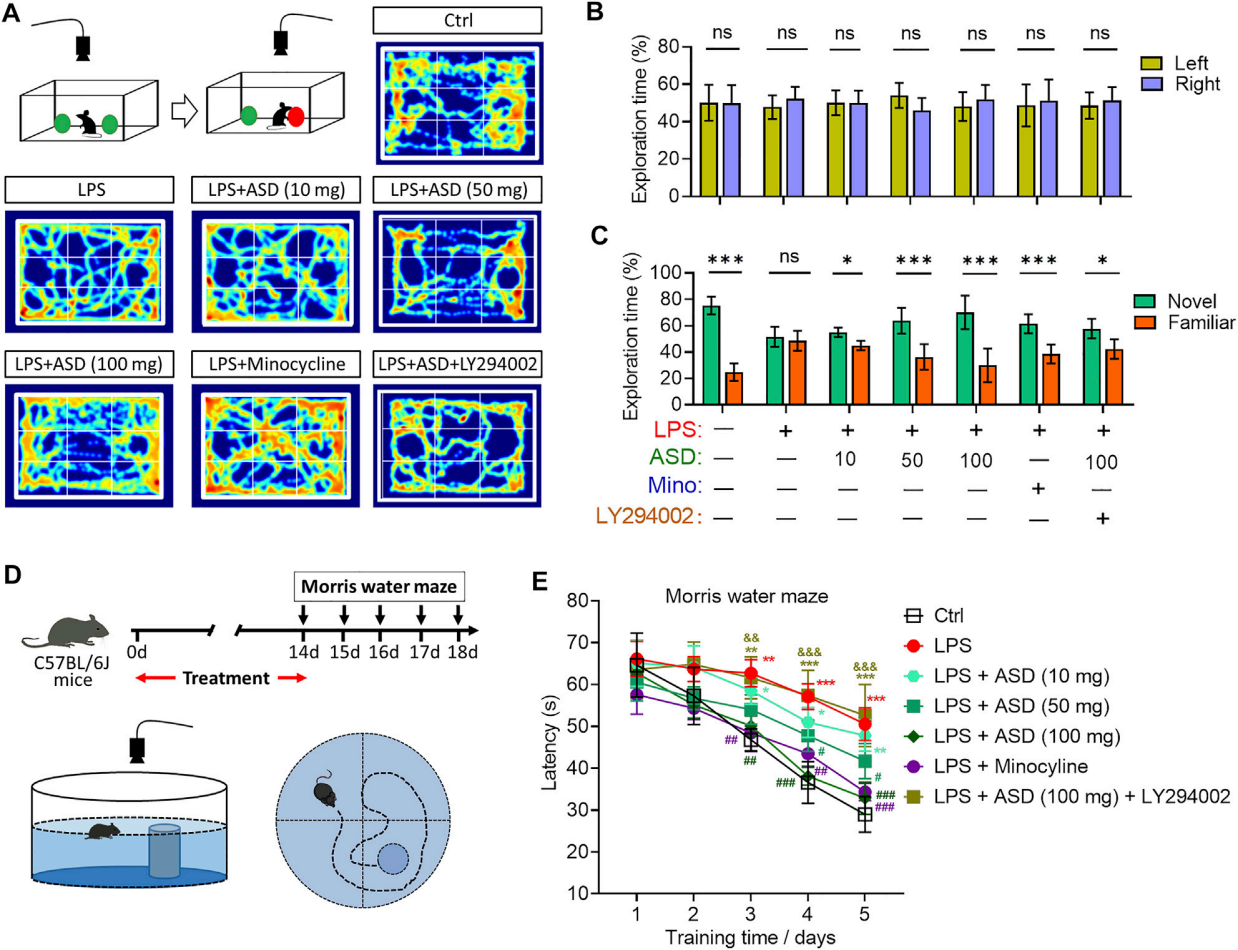
Effects of ASD on cognitive impairment of mice chronically exposed to LPS in the presence or absence of PI3K-Akt inhibitor. **(A)**, Scheme describing the experimental evaluation of mice using the novel object recognition test, and heatmap of mouse behavior in this test. **(B)**, Mice showed a preference for the novel item, regardless of whether it replaced the familiar object on the right or left. **(C)**, Comparison of preference for the novel or familiar object. **(D)**, Scheme describing the experimental evaluation of learning and memory using the Morris water maze test. **(E)**, Morris water maze latency of mice treated with saline (Ctrl) or lipopolysaccharide (LPS), followed by akebia saponin D (ASD), minocycline or PI3K-Akt inhibitor (LY294002). Data are mean ± standard error of the mean (SEM) (n = 8-11). Panels (B) and (C): *p < 0.05, ***p < 0.001 vs. left (B) or novel (C) by a paired Student’s t-test. Panel (E): *p < 0.05, **p < 0.01, ***p < 0.001 vs. Ctrl group, #*p* < 0.05, ##*p* < 0.01, ###*p* < 0.001 vs. LPS group, &&*p* < 0.01, &&&*p* < 0.001 vs. ASD (100 mg/kg) + LPS group by one-way ANOVA with Tukey’s multiple-comparisons test.

Cognition was tested through the novel object recognition test (Figure 10A). No preference was shown for the novel item, regardless of whether it replaced the familiar object on the right or left (Figure 10B). Chronic exposure to LPS induced a significant cognitive defect (Figure 10C), which ASD (at 10–100 mg/kg/d) or minocyline ameliorated (Figures 10A–C).

Learning and memory were assessed using a Morris water maze (Figure 10D). Chronic exposure to LPS prolonged latency time in the maze, which ASD (at a dose of at least 50 mg/kg/d) or minocyline reversed (Figure 10E).

**FIGURE 11:**
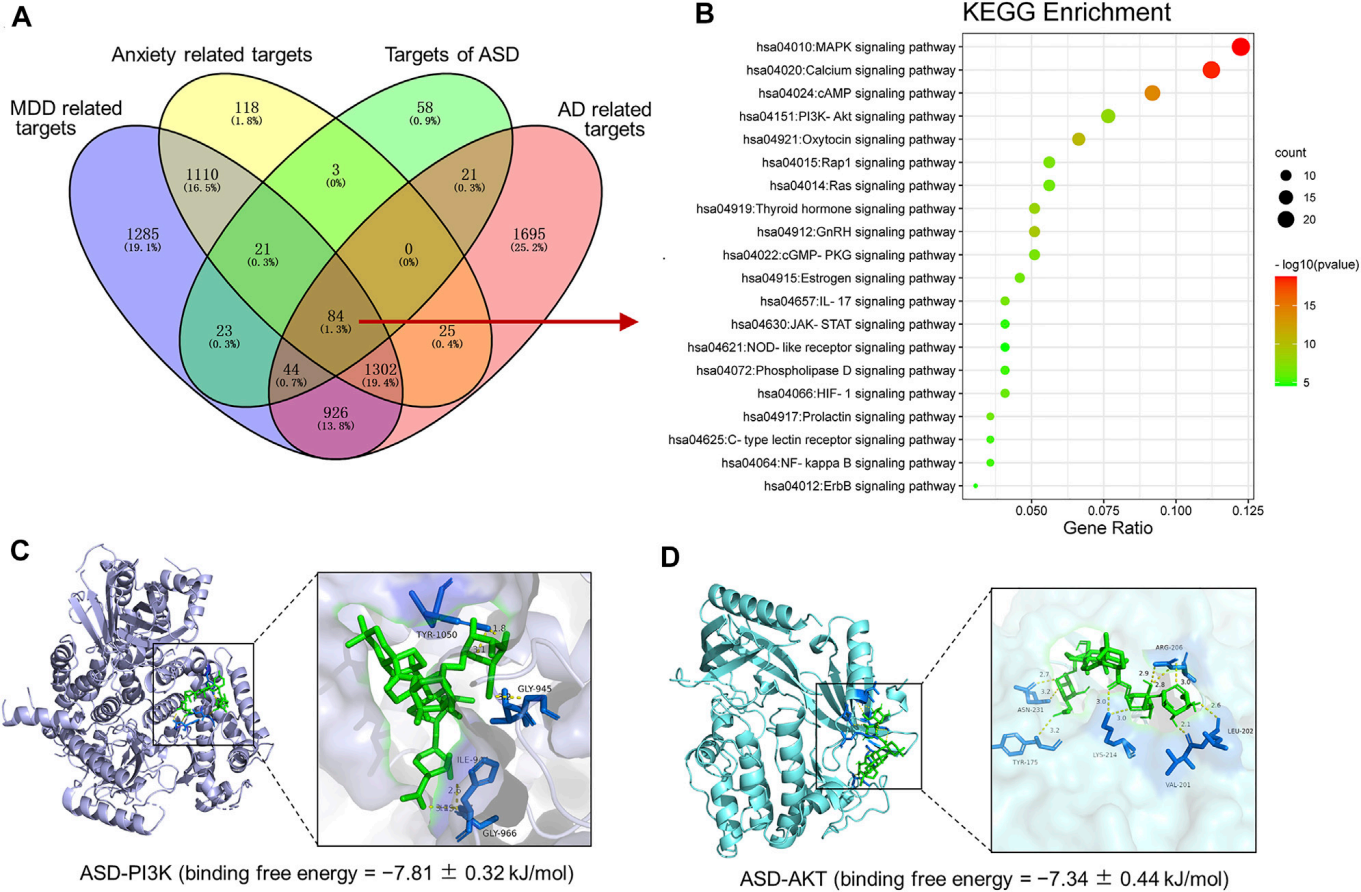
Pharmacological mechanisms of ASD against disorders involving impaired neurogenesis. **(A)**, Venn diagram summarizing the intersection targets of the akebia saponin D (ASD), major depressive disorder (MDD), anxiety and Alzheimer’s disease (AD). **(B)**, KEGG analysis of key targets of ASD in treatment of MDD, anxiety and AD. Bubble plot of top 20 KEGG pathways. **(C,D)**, Molecular docking diagram of ASD (green) to target PI3K (C) and Akt (D). The yellow lines represent the hydrogen bond interaction force, which is the main force promoting molecule binding with the active site. The blue positions indicate the amino acid residues of the receptors (PI3K/AKT).

To further expound the pharmacological mechanisms of ASD against disorders involving impaired neurogenesis, we performed network pharmacology-based analysis of ASD for the treatment of major depressive disorder (MDD), anxiety and Alzheimer’s disease (AD). We identified 4795 MDD-related targets, 2663 AD-related targets, 4097 AD-related targets, 2,663 anxiety-related targets (Supplementary Figure S2). A total of 1,386 targets involving MDD, AD and anxiety were identified, of which 84 were ASD targets (Figure 11A). These common targets were extracted for further Kyoto Encyclopedia of Genes and Genomes (KEGG) pathway enrichment analysis using R software. The results showed that the PI3K-Akt pathway ranks fourth in KEGG pathway enrichment (Figure 11B).

To probe the possible binding mode of ASD with the PI3K/Akt, molecular docking was performed. ASD is bound to the interaction interface of the PI3K and AKT (Figures 11C,D). Upon calculation of the MM/GBSA binding energy, the ligand ASD bound to the PI3K with a stability of −7.81 ± 0.32 kJ/mol (Figure 11C); and the ligand ASD bound to the AKT with a stability of −7.34 ± 0.44 kJ/mol (Figure 11D).

In support of PI3K-Akt signaling as mediator of the neuroprotective effects of ASD, PI3K-Akt inhibitor LY294002 blocked the compound’s ability to reverse the LPS-induced defects on all these behavioral tests (Figures 8A,10E). These results suggested that PI3K-Akt pathway is one of the targets of ASD to against the disorders involving impaired neurogenesis.

## 4 Discussion

Neurogenesis plays a fundamental role in the postnatal brain, where it is required for neuronal plasticity (Puderbaugh & Emmady, 2022). Promoting neurogenesis in the adult brain may be an effective strategy for treating major depressive disorder and Alzheimer’s disease, and the compound ASD from traditional Chinese medicine has shown promise in this regard. In the present work, we found that ASD can promote NSPC proliferation, survival and neuronal differentiation *in vitro* and *in vivo*, even in a microglia-mediated inflammatory niche. These effects correlate with milder behavioral symptoms of depression, anxiety and cognitive impairment due to chronic neuroinflammation. Finally, we provide evidence that the therapeutic effects of ASD involve activation of the PI3K-AKT pathway. To our knowledge, this study is the first to report the direct regulation of ASD on adult hippocampal NSPCs.

The hippocampus, a brain area critical for learning, memory and emotion, is especially vulnerable to damage in early stages of AD, chronic stress or inflammation, when neurogenesis in the adult hippocampus is altered (Zonis et al., 2015; Chesnokova et al., 2016; Babcock et al., 2021; Du Preez et al., 2021). Various key molecules involved in the pathogenesis of major depression disorder or AD have been linked to the inhibition of neurogenesis (Berger et al., 2020; Babcock et al., 2021; Walgrave et al., 2021). Brain imaging and postmortem studies of patients with either of these disorders indicate reduction in hippocampal volume perhaps due to reduced neurogenesis and loss of mature neurons (Frimodt-Møller et al., 2019; Zarate-Garza et al., 2021).

This reduced neurogenesis seems to be at least partly the fault of microglia. These immune cells create an inflammatory microenvironment that inhibits NSPC proliferation and differentiation (Xiong et al., 2016; Zhang et al., 2020b). In a variety of neurodegenerative settings, microglia alter their transcriptional profile, morphology, and function to exert negative effects in disease models (Mattei et al., 2017; Wolf et al., 2017). Activation of microglia results in phagocytosis and production of pro-inflammatory cytokines, reactive oxygen species, and inducible NO synthase (iNOS), which can alter the hippocampal neurogenic niche, reducing NSPC proliferation, survival and neuronal differentiation. These injuries can contribute to cognitive dysfunction and depression (Snyder et al., 2011; Berger et al., 2020; Zhang et al., 2020b; Zhang J. et al., 2021; Mizuno et al., 2021). In the present study, we found that mice chronically exposed to LPS exhibited obvious depressive- and anxiety-like behaviors and cognitive impairment, which were accompanied by microglial overactivation and inhibition of hippocampal neurogenesis. These results support the idea that microglia-mediated neuroinflammation inhibits hippocampal neurogenesis, with diverse behavioral consequences related to depression, anxiety and cognitive impairment.

Adult hippocampal neurogenesis is supported by NSPC proliferation and neuronal differentiation, as well as maturation and survival of the resulting newborn neurons (Deierborg et al., 2010; Pilz et al., 2018). These processes are strongly dependent on a supportive hippocampal neurogenic niche, which activated microglia antagonize by creating an inflammatory microenvironment (Toda et al., 2019; Zhang et al., 2020b). Consistent with this, we found that chronic LPS exposure reduced the numbers of BrdU + cells, BrdU + -DCX + cells and BrdU + -NeuN + cells in mouse hippocampus. Indeed, conditioned medium from microglia treated with LPS was sufficient to induce NSPC apoptosis and suppress their proliferation and neuronal differentiation.

Several studies have shown that inhibitors of microglial activation such as minocycline can suppress pro-inflammatory cytokine expression and restore hippocampal neurogenesis, as well as ameliorate depressive-like behaviors and cognitive impairment (Zhang et al., 2020b; Alavi et al., 2021; Bassett et al., 2021). Our results are consistent with the observation that variations in the relatively small number of new neurons in adult brains of humans and rodents is strongly linked to the pathogenesis and remission of neuropsychiatric disorders (Grace et al., 2021). We show here that ASD exerted therapeutic effects similar to those of minocycline, yet it did not reverse the LPS-induced increase in the area of Iba1+ cells or IL-1β level. Thus, it appears that ASD restores hippocampal NSPC proliferation, survival and neuronal differentiation, as well as the synaptic function of hippocampal neurons in a microenvironment of chronic neuroinflammation. However, different from what we found in chronic mild stress model mice (Jiang et al., 2022), ASD does not appear to completely inhibit microglia-mediated inflammation in hippocampus of chronic LPS-treated mice as minocycline does. Instead, ASD acts directly on NSPCs to promote their proliferation, survival and neuronal differentiation. It is interesting that ASD at 100 mg/kg/d for 14 days promoted the proliferation of NSPCs in normal mice, but did not affect hippocampal neurogenesis and the maturation of newborn neurons. It may be that the healthy body itself monitors neurogenesis and limits the number of newborn neurons by inducing apoptosis of excess immature neurons.

Inhibited neurogenesis may exacerbate neuronal vulnerability to Alzheimer’s disease, stress or immune challenge, leading to memory impairment, anxiety or depression; under these conditions, enhanced neurogenesis may be a compensatory response to repair and protect the brain (Bassani et al., 2018; Moreno-Jiménez et al., 2019; Tobin et al., 2019). Adult neurogenesis in the hippocampus subgranular zone (SGZ) is associated with the etiology and efficacy of treatments against Alzheimer’s disease and major depressive disorder (Anacker & Hen, 2017; Babcock et al., 2021; Zhang J. et al., 2021). Adult hippocampal neurogenesis appears to occur in humans as well as in rodents, although this idea is still controversial (Spalding et al., 2013; Kempermann et al., 2018; Moreno-Jiménez et al., 2021). In this study, we isolated NSPCs from the hippocampal subgranular zone of adult mice and induced their proliferation and differentiation, suggesting that NSPCs do indeed exist in the adult hippocampus of rodents.

The PI3K/Akt signaling pathway is the classical anti-apoptotic and pro-survival signal transduction pathway (Gabbouj et al., 2019; Xie et al., 2019). When PI3K binds to growth factor receptors such as the epidermal growth factor receptor, Akt becomes activated, leading to the activation or inhibition of downstream substrates such as the apoptotic proteins Bad and Caspase-9 (Jeong et al., 2008). These substrates go on to regulate cell proliferation, differentiation, apoptosis, migration, and other processes (Darici et al., 2020). Base on the network pharmacology analysis and molecular docking, we found PI3K-Akt pathway is one of the targets of ASD to against the disorders involving impaired neurogenesis, such as Alzheimer’s disease, major depressive disorder and anxiety disorder. In our mouse model of chronic neuroinflammation, levels of PI3K and pAkt were strikingly reduced in hippocampus, and this was associated with reduced NSPC proliferation and differentiation. The PI3K-Akt signaling pathway in adult NSPCs is strongly inhibited in an environment of neuroinflammation mediated by microglia, leading to increased apoptosis and differentiation into astrocytes. Conversely, ASD strongly increased levels of PI3K, Akt, and pAkt proteins, even in the presence of LPS-M-CM. These results suggest that the PI3K-Akt pathway mediates the effects of ASD on NSPC proliferation, survival and neuronal differentiation. Consistent with this, we found that LY294002, an inhibitor of the PI3K/Akt pathway, blocked the effects of ASD on NSPC apoptosis, proliferation and neuronal differentiation in the presence or absence of LPS-M-CM. LY294002 treatment also blocked the effects of ASD on hippocampal neurogenesis and depressive- and anxiety-like behaviors and cognitive impairment. Our results add to the number of cellular contexts in which ASD activates PI3K/Akt signaling with therapeutic effects, including bone regeneration (Ke et al., 2016) as well as Alzheimer’s disease (Zheng et al., 2017; Razani et al., 2021), anxiety (Qiao et al., 2018) and depression (Guo et al., 2019; Shan et al., 2020). This growing literature argues for activating PI3K/Akt signaling as a strategy against neurological disorders involving impaired neurogenesis.

**FIGURE 12:**
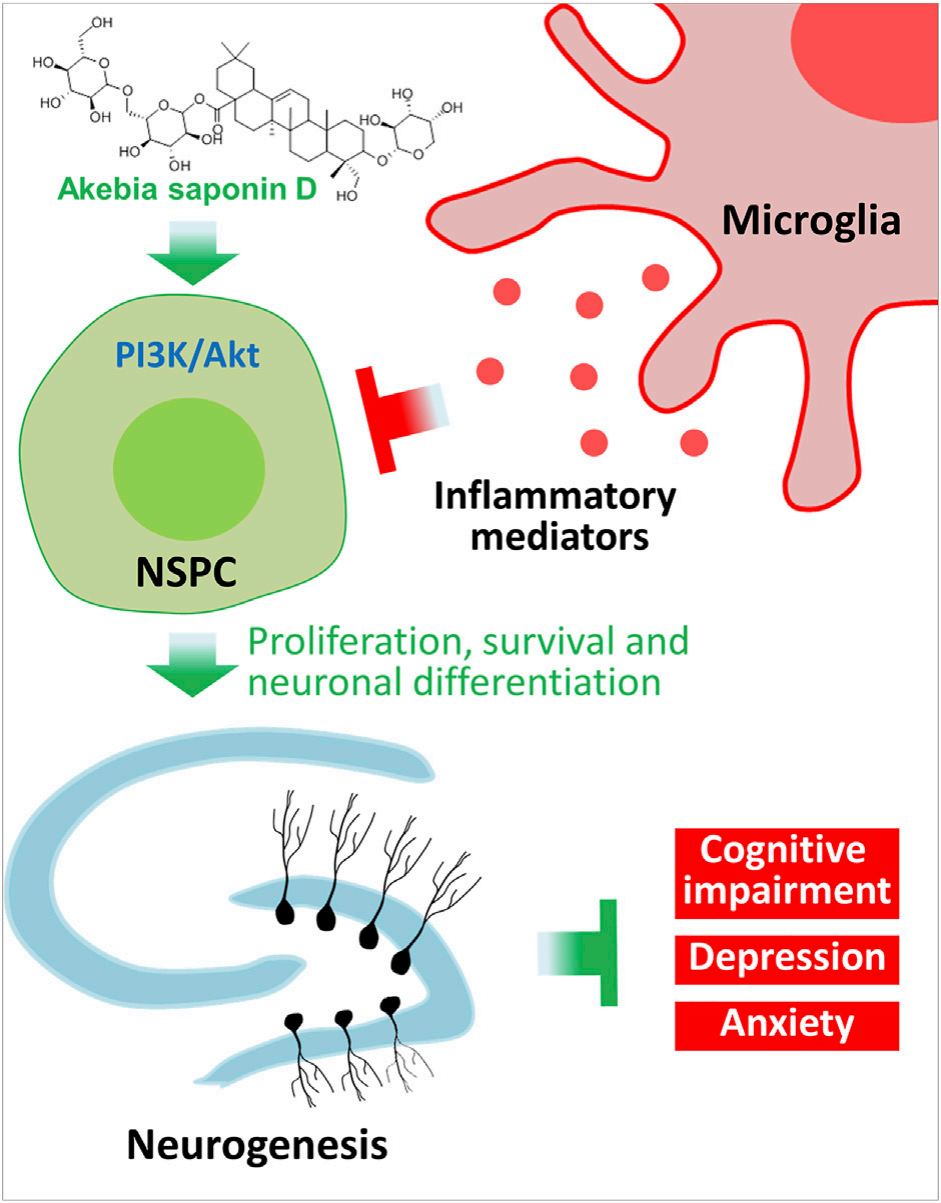
Schematic diagram of how akebia saponin D may protect hippocampal neurogenesis. Akebia saponin D activates the PI3K-Akt pathway to protect NSPCs from the microglia-mediated inflammatory niche, promoting their proliferation, survival and neuronal differentiation, as well as ameliorating depressive- and anxiety-like behaviors and cognitive impairment.

Our results provide strong evidence that ASD protects NSPCs from the microglia-mediated inflammatory niche and promotes their proliferation, survival and neuronal differentiation by activating the PI3K-Akt pathway (Figure 12). Our results support further evaluation of ASD for the treatment of Alzheimer’s disease, major depressive disorder and other conditions associated with impaired neurogenesis.

## Data Availability

The original contributions presented in the study are included in the article/Supplementary Material, further inquiries can be directed to the corresponding authors.

## Abbreviations

AD, Alzheimer’s disease; ASD, Akebia saponin D; BDNF, brain-derived neurotrophic factor; BrdU, 5′-bromo-2′deoxyuridine; DAPI, 4′,6-diamidino-2-phenylindole; DCX, doublecortin; DG, dentate gyrus; GCL, granular cell layer; GFAP, glial fibrillary acidic protein; Iba1, ionized calcium binding adapter molecule 1; MAP2, Microtubule-associated protein 2; MDD, major depressive disorder; NeuN, neuron-specific nucleoprotein; NSPC, neural stem/progenitor cell; PBS, phosphate-buffered saline; PI3K/AKT, phosphatidylinositol 3 kinase/serine-threonine kinase; LPS, lipopolysaccharide; SGZ, subgranular zone.
