# Supplementary material for: Akebia saponin D protects hippocampal neurogenesis from microglia-mediated inflammation and ameliorates depressive-like behaviors and cognitive impairment in mice through the PI3K-Akt pathway
Source: Front Pharmacol. 2022 Aug 30;13:927419. doi: 10.3389/fphar.2022.927419 (PMC9468712; doi:10.3389/fphar.2022.927419)
Supplement: Supplementary file 1 [file DataSheet1.PDF]

Supplementary figures and legends

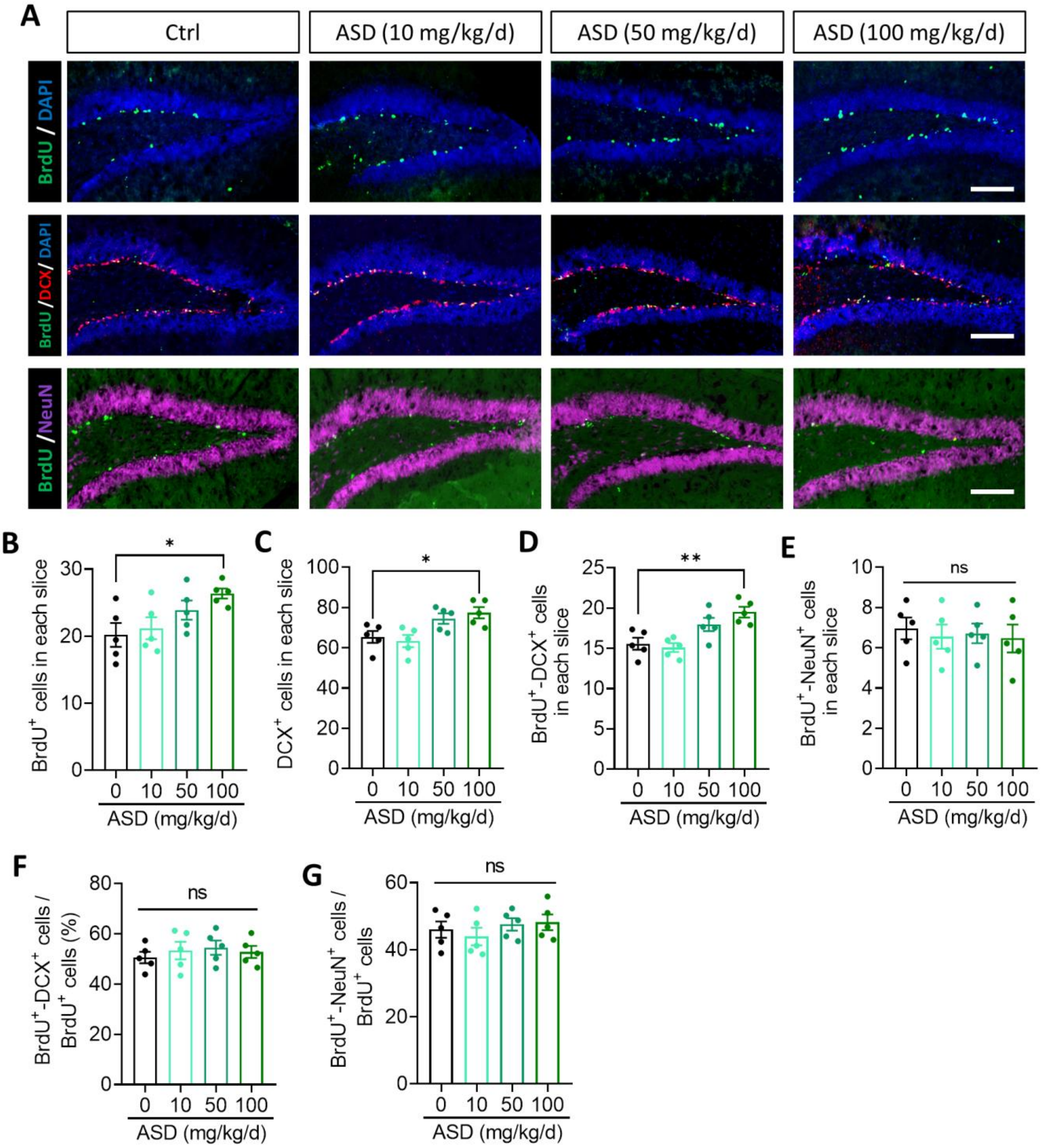

Figure S1 Effects of ASD on NSPC proliferation and differentiation and maturation of newborn neurons in dentate gyrus of normal mice.

A, Immunofluorescence micrographs of BrdU$^+$ cells, BrdU$^+$-DCX$^+$ cells and BrdU$^+$-NeuN$^+$ cells in the DG of normal mice treated with ASD or saline. Proliferating NSPCs were labelled with BrdU (green); immature neurons, with antibody against doublecortin (DCX); and newborn neurons differentiated from NSPCs, with both BrdU and anti-DCX antibody (white arrowheads). Mature neurons were labeled with

antibody against neuron-specific nucleoprotein (NeuN); and mature neurons differentiated from NSPCs, with both BrdU and anti-NeuN antibody. Scale bar, 100 μm.

B-E, Quantification of the hippocampal BrdU^{+} cells, DCX^{+} cells, BrdU^{+}-DCX^{+} cells and BrdU^{+}-NeuN^{+} cells in each slice.

F, Quantification of the percentage of total BrdU^{+} cells in DG that were BrdU^{+}-DCX^{+} cells.

G, Quantification of the percentage of total BrdU^{+} cells in the DG that were BrdU^{+}-NeuN^{+} cells.

Five mice from each group were examined, and five hippocampal micrographs (40×) from each animal were quantified. Each dot in the bar graph represents the average of all micrographs for each mouse. Data are mean ± standard error of the mean (SEM) (n=5), *P < 0.05, **P < 0.01 vs. Ctrl group by one-way ANOVA with Tukey's multiple-comparisons test. Each dot in the bar graph represents the average of all micrographs for each mouse.

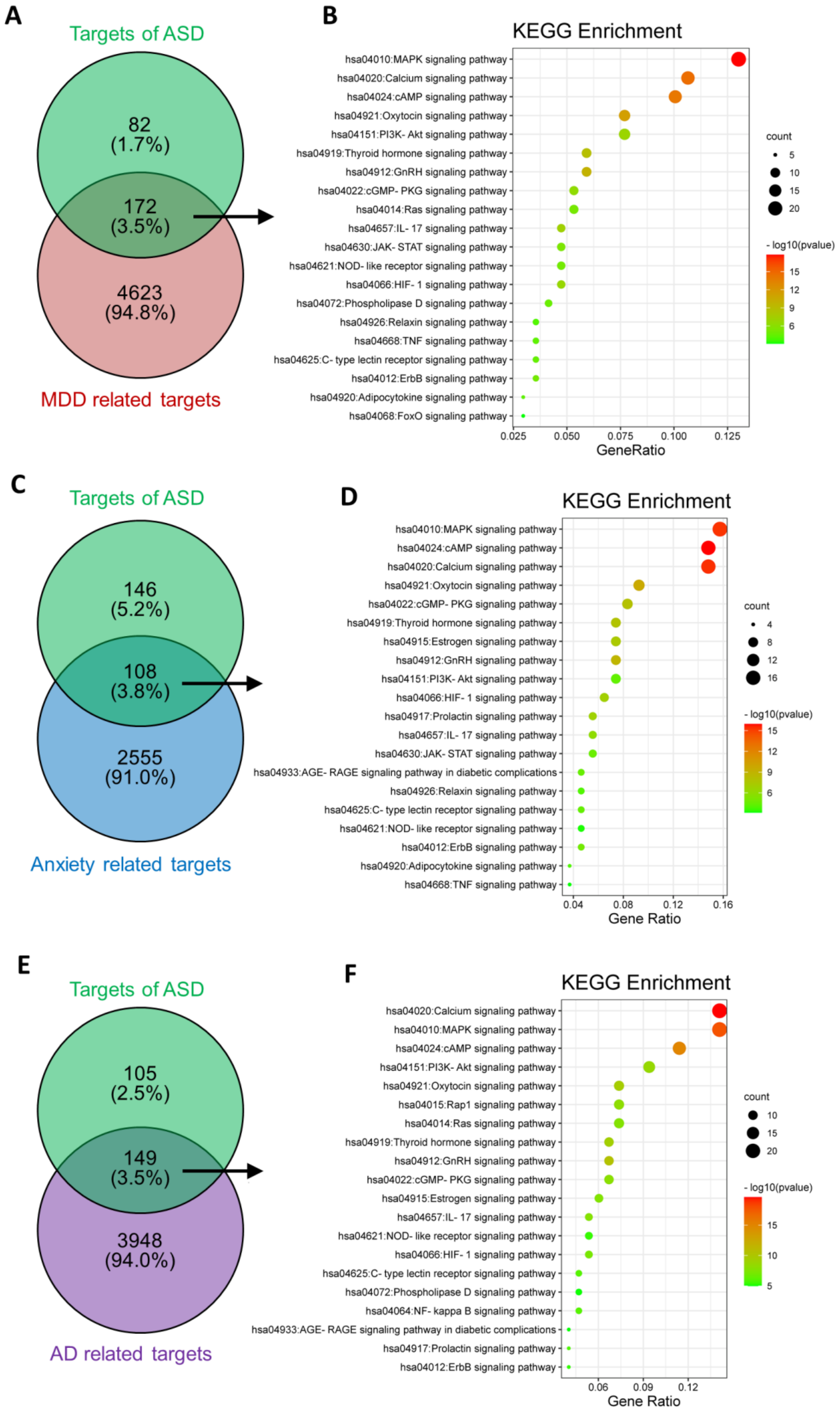

Fig. S2 Network pharmacology analysis of ASD against major depressive disorder, anxiety and Alzheimer's disease.

A, Venn diagram summarizing the intersection targets of the akebia saponin D (ASD) and major depressive disorder (MDD).

B, KEGG analysis of key targets of ASD in treatment of MDD. Bubble plot of top 20 KEGG pathways.

C, Venn diagram summarizing the intersection targets of the ASD and anxiety.

D, KEGG analysis of key targets of ASD in treatment of anxiety. Bubble plot of top 20 KEGG pathways.

E, Venn diagram summarizing the intersection targets of the ASD and AD.

F, KEGG analysis of key targets of ASD in treatment of AD. Bubble plot of top 20 KEGG pathways.
